# Single-Molecule Peptide Identification Using Fluorescence Blinking Fingerprints

**DOI:** 10.1021/jacs.2c12561

**Published:** 2023-01-05

**Authors:** Salome Püntener, Pablo Rivera-Fuentes

**Affiliations:** † Institute of Chemical Sciences and Engineering, Ecole Polytechnique Fédéral de Lausanne, CH-1015 Lausanne, Switzerland; ‡ Department of Chemistry, University of Zurich, CH-8057 Zurich, Switzerland

## Abstract

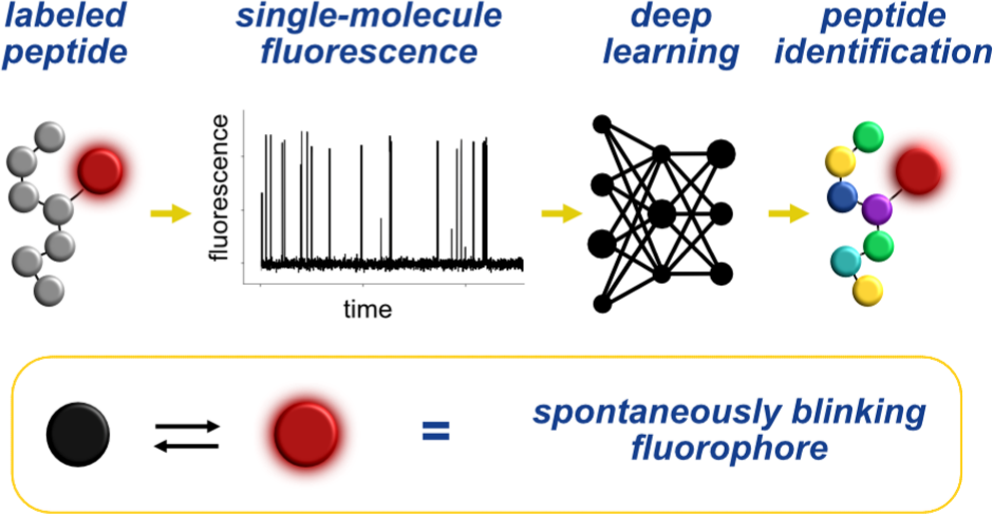

The ability to identify peptides with single-molecule sensitivity would lead to next-generation proteomics methods for basic research and clinical applications. Existing single-molecule peptide sequencing methods can read some amino acid sequences, but they are limited in their ability to distinguish between similar amino acids or post-translational modifications. Here, we demonstrate that the fluorescence intermittency of a peptide labeled with a spontaneously blinking fluorophore contains information about the structure of the peptide. Using a deep learning algorithm, this single-molecule blinking pattern can be used to identify the peptide. This method can distinguish between peptides with different sequences, peptides with the same sequence but different phosphorylation patterns, and even peptides that differ only by the presence of epimerized residues. This study builds the foundation for a targeted proteomics method with single-molecule sensitivity.

## Introduction

The diversity of the proteome is only partially determined by the genome. Protein abundance, the occurrence of isoforms, and post-translational modifications (PTMs) cannot be predicted from genomic or transcriptomic information. Mass spectrometry-based proteomics methods^1,2^ remain limited in their sensitivity and dynamic range compared to single-molecule approaches, which are now well established in nucleic acid analysis.^3^ In particular, single-molecule identification of peptides and proteins would enable the analysis of biomarkers that are present in very small quantities, for example, in diluted clinical samples, single cells, or isolated organelles.^4^

Recently, significant progress has been made toward single-molecule proteomics.^4,5^ Approaches based on biological^6−11^ and solid-state nanopores,^12^ tunneling conductance measurements,^13^ N-terminal amino-acid-binding probes,^14^ single-molecule Edman sequencing,^15^ DNA nanotechnologies,^16,17^ and mass spectrometry^18^ have been reported. All of these methods hold great potential, but they also face several challenges. Nanopore-based sequencing struggles with amino acid detection accuracy, linearization of large peptides and proteins, translocation of positively charged peptides, and throughput. Single-molecule fluorescence-based techniques are massively parallelizable, but some of these methods rely on multiple cycles of chemical or enzymatic degradation,^14,15^ making data acquisition long and prone to errors. Other fluorescence-based methods can detect accurately the position of selected amino acids,^19,20^ but it is unclear how sensitive these methods are to the nature of PTMs present in other amino acids in the peptide. Here, we provide proof of a fundamentally new approach to identifying single-peptide molecules. Similar to other fingerprinting methods, our approach is not based on sequencing. Therefore, it avoids the need to read the peptide sequence and distinguish each amino acid with high accuracy in order to recognize a known target peptide. This technique holds potential as a widely applicable and accurate single-molecule peptide fingerprinting technology.

## Results and Discussion

**Figure 1:**
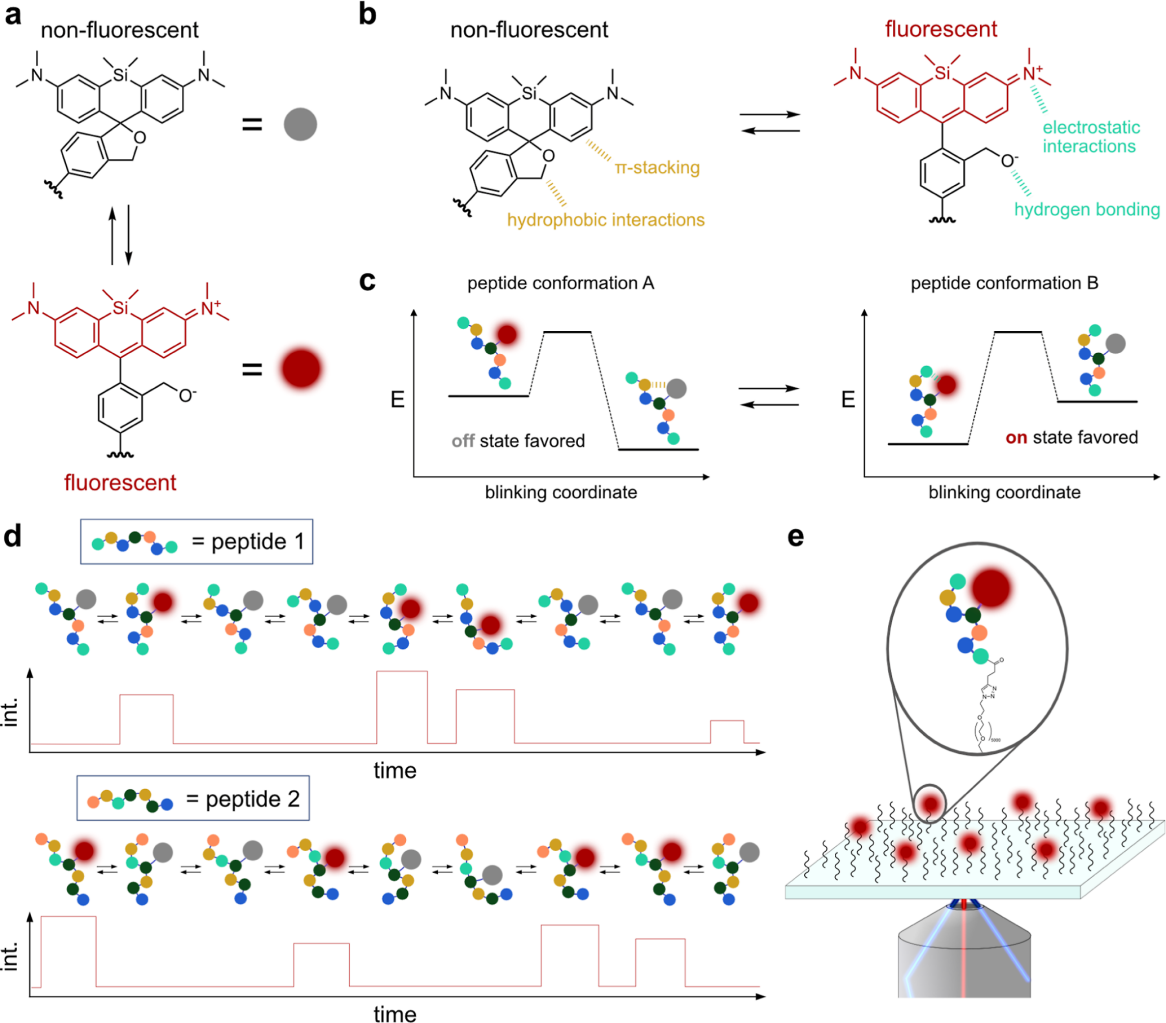
Blinkognition concept. (a) Chemical structure of the spontaneously blinking fluorophore used here (HMSiR).^21^ (b) Interactions that could stabilize either the fluorescent or non-fluorescent isomer of HMSiR. (c) Different conformations of the peptide could lead to interactions with certain amino acids that stabilize either the fluorescent or the non-fluorescent isomer of HMSiR. (d) The blinking pattern (intensity over time) of the fluorophore should be different when it is attached to different peptides. (e) Peptide–fluorophore conjugates are attached via click chemistry to a PEGylated glass surface and imaged using TIRF microscopy.

Spontaneously blinking fluorophores have been used for single-molecule localization microscopy because they undergo a ground-state isomerization between a fluorescent and a non-fluorescent isomer, producing an intermittent pattern of emission (spontaneous blinking).^21−24^ Hydroxymethyl silicon rhodamine (HMSiR) is a prototypical example of a spontaneously blinking fluorophore.^21^ This dye isomerizes in the ground state between a non-fluorescent spirocyclic and a fluorescent zwitterionic form with a low barrier of interconversion (Figure 1a). Because the isomerization occurs in the ground state, the blinking behavior of HMSiR is largely independent of the excitation power. The fluorescent and non-fluorescent isomers of HMSiR differ from each other in terms of charge, polarity, hydrogen-bonding ability, π-surface, and so forth. Thus, their relative stabilities and barriers of interconversion can be expected to be affected by factors such as solvation, hydrogen bonding, electrostatic and hydrophobic interactions, and so forth (Figure 1b). Such interactions have also been demonstrated to affect the kinetics of photoinduced processes, which is reflected in single-molecule blinking behaviors.^25,26^ We reasoned that when HMSiR is covalently attached to a peptide, these interactions are mostly provided by the side chains of the constituent amino acids, and therefore, different conformations of the peptide would stabilize either the fluorescent or the non-fluorescent isomer of HMSiR to different extents (Figure 1c). Over time, the dynamic interaction of HMSiR with the peptide would lead to a certain pattern of fluorescent and non-fluorescent states (blinking pattern). Peptides containing different amino acids would have different blinking patterns (Figure 1d). We hypothesized that even if these blinking patterns were too complex to be predicted ab initio, we could record them experimentally from pure synthetic samples attached to a glass surface and imaged by total internal reflection fluorescence (TIRF) microscopy (Figure 1e). By recording enough blinking traces, a machine learning (ML) model could be trained on this synthetic ground truth to recognize a specific peptide in a mixture. We call this approach “blinkognition”, a portmanteau of “blinking” and “recognition”.

**Figure 2:**
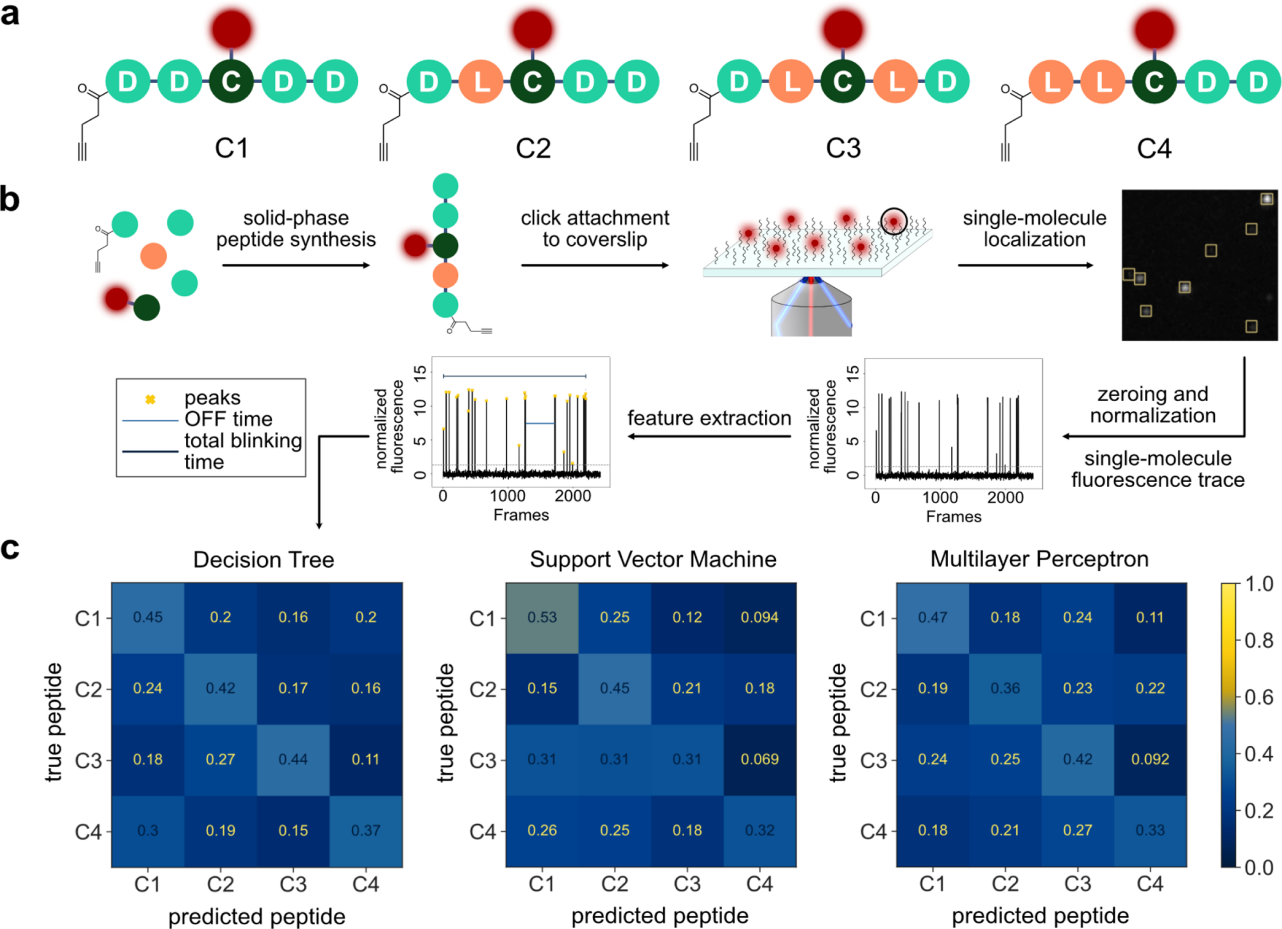
Blinking pattern acquisition and analysis pipeline. (a) Structures of peptides **C1–C4** indicating the blinking fluorophore (red circle) attached to the cysteine (C) residue and the N-terminal alkyne for surface conjugation. (b) Workflow used to obtain the datasets for classification experiments (for details, see the Supporting Methods). (c) Confusion matrices for the classification achieved with the supervised ML models using the features extracted from 479 traces (test set) of **C1–C4**.

To test the blinkognition hypothesis, we first examined whether we could accurately distinguish peptides within a set of four, highly similar, negatively charged peptides (**C1–C4**, Figure 2a) based on the spontaneous blinking of an HMSiR fluorophore (Figure 1a) covalently attached to a cysteine (C) residue within the peptide. The choice of cysteine as the residue to be modified is arbitrary, and the method could be applied to any amino acid that can be bioconjugated.

Peptides **C1–C4** were synthesized by solid-phase peptide synthesis, and glass coverslips were cleaned with ozone and passivated with a mixture of polyethylene glycol (PEG) and PEG with a terminal azide (Supporting Methods). Each peptide was attached to a separate coverslip by click chemistry and imaged using TIRF microscopy (Figure 2b, Supporting Methods, and Figures S1–S3). For all peptides, the imaging buffer, temperature, surface passivation method, excitation power, exposure time, and total imaging time were kept constant across multiple replicates (separate coverslips prepared and measured on different days). Single-molecule fluorescence time traces were extracted from time-lapse acquisitions, and the intensity of each trace was normalized (Figure 2b, Supporting Methods, Figures S4–S6, and Tables S1 and S2). We labeled the traces according to the peptide that they belong to and combined them in a dataset containing traces and labels. We split this dataset into train and test sets (80/20) and manually extracted a few features from the traces, including the total number of peaks, peak duration, and photobleaching time (a full list of features is provided in Table S3). However, visualization methods, for example, correlation plots (Figure S7), did not reveal obvious clustering for the different peptides. Similarly, we could not distinguish between peptides by carrying out principal component analysis (PCA, Figure S8). Fourier-transformed traces did not reveal any easily recognizable patterns either.

Given that unsupervised methods could not distinguish between peptides **C1–C4**, we used the features extracted from traces in the training set to train supervised ML models and evaluated them on the traces in the test set. These models could identify the molecules only with modest accuracies (35–45%, Figure 2c, Supporting Methods, Table S4, and Figure S9), albeit significantly higher than a random guess (25%). Although these results are not accurate enough for practical applications, they demonstrate that interpretable ML models can extract some sequence-related information from blinking patterns, suggesting that blinkognition is a viable strategy for single-molecule peptide fingerprinting.

**Figure 3:**
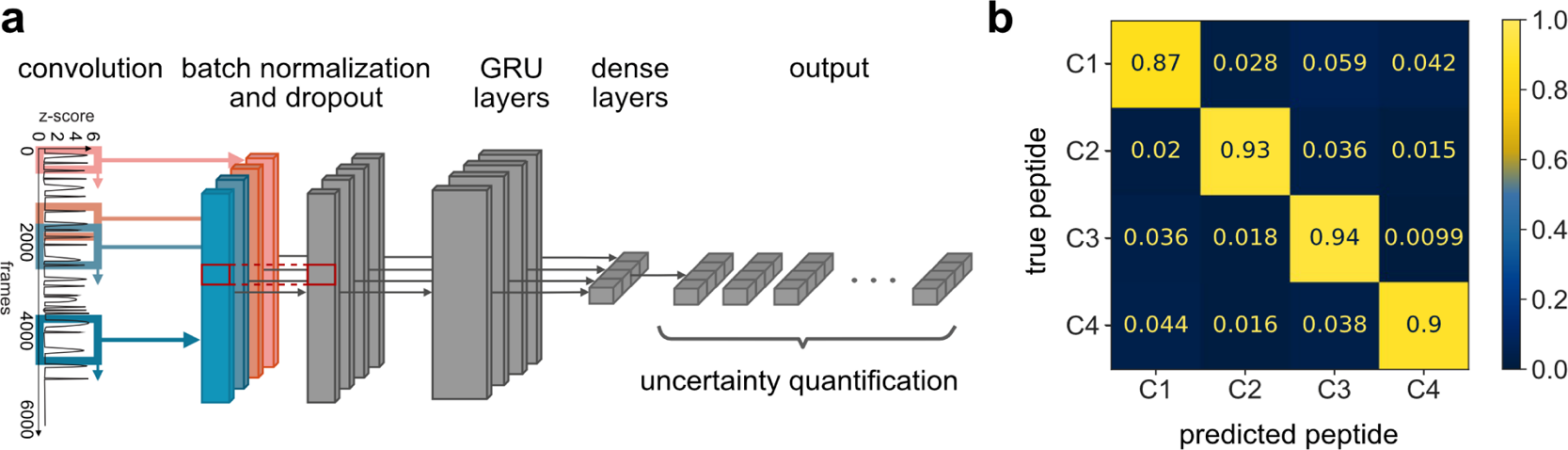
Peptide identification by blinkognition using a deep learning model. (a) Schematic depiction of the architecture used for the deep learning classification approach. The normalized traces are directly used as an input for the convolutional layers with batch normalization and dropout layers interspersed. The output of the convolutional layers is fed into GRU layers, followed by a dense and softmax layer that produces the classification output. (b) Confusion matrix for the classification achieved with the model described in (a) of 1208 peptide traces of **C1–C4**.

We posited that our manually extracted features might not contain all the information that contributes to the uniqueness of blinking traces. Thus, we designed a deep learning classifier that utilizes a one-dimensional convolutional neural network (1D-CNN) for downsampling and feature extraction directly from normalized blinking traces. The accuracy of a purely convolutional model, however, remained within the range of classical ML algorithms (40–50%, Figure S10). We hypothesized that it could be beneficial to add layers to the model that enabled it to retain information along the time coordinate, potentially over the entire acquisition. This issue has been addressed in ML by using recurrent neural networks (RNNs). Such architectures contain loops that retain previous information if it is relevant at a later time point. They have been extensively used in language models and machine translation to allow models to learn words in context.^27^ Gated recurrent units (GRUs) are a subtype of RNNs that can learn and remember long-term information in a sequence but also “forget” irrelevant input along the sequence. Compared to similar architectures (e.g., long short-term memory cells), GRUs are computationally more efficient and are reported to require less training data.^27,28^ Therefore, we added GRU layers after the CNN layers to our model. The output is subsequently fed into the fully connected layers that produce a classification output (Figure 3a, Supporting Methods, Tables S5–S8, and Figure S5). The robustness of the 1D-CNN–GRU architecture was tested by nested cross-validation (Supporting Methods and Figure S11). Furthermore, we implemented Monte Carlo dropout during inference to filter out low-quality traces based on the certainty of classification (Supporting Methods, Table S9, and Figure S12).^29^ Using this deep learning architecture, we achieved identification accuracies of ∼90% for peptides **C1–C4** (Figure 3b). Additionally, to ensure that the deep learning algorithm is learning information that is related to the blinking pattern and not just noise in the traces, we extracted the signal from regions of the TIRF movies that did not contain any molecules and labeled them as peptides. The deep learning model failed to learn when it was trained on these noise signals and produced only random predictions with high uncertainty (Figure S13). Additionally, we also used the traces obtained from real molecules but scrambled their labels for training. The deep learning model also failed to learn when it was trained on this scrambled dataset and could not predict the identities of molecules in an unscrambled test set. These control experiments indicate that, although the blinking patterns of different peptides look very similar to the human brain (Figure S6), our deep learning model learns to distinguish between them with high accuracy.

**Figure 4:**
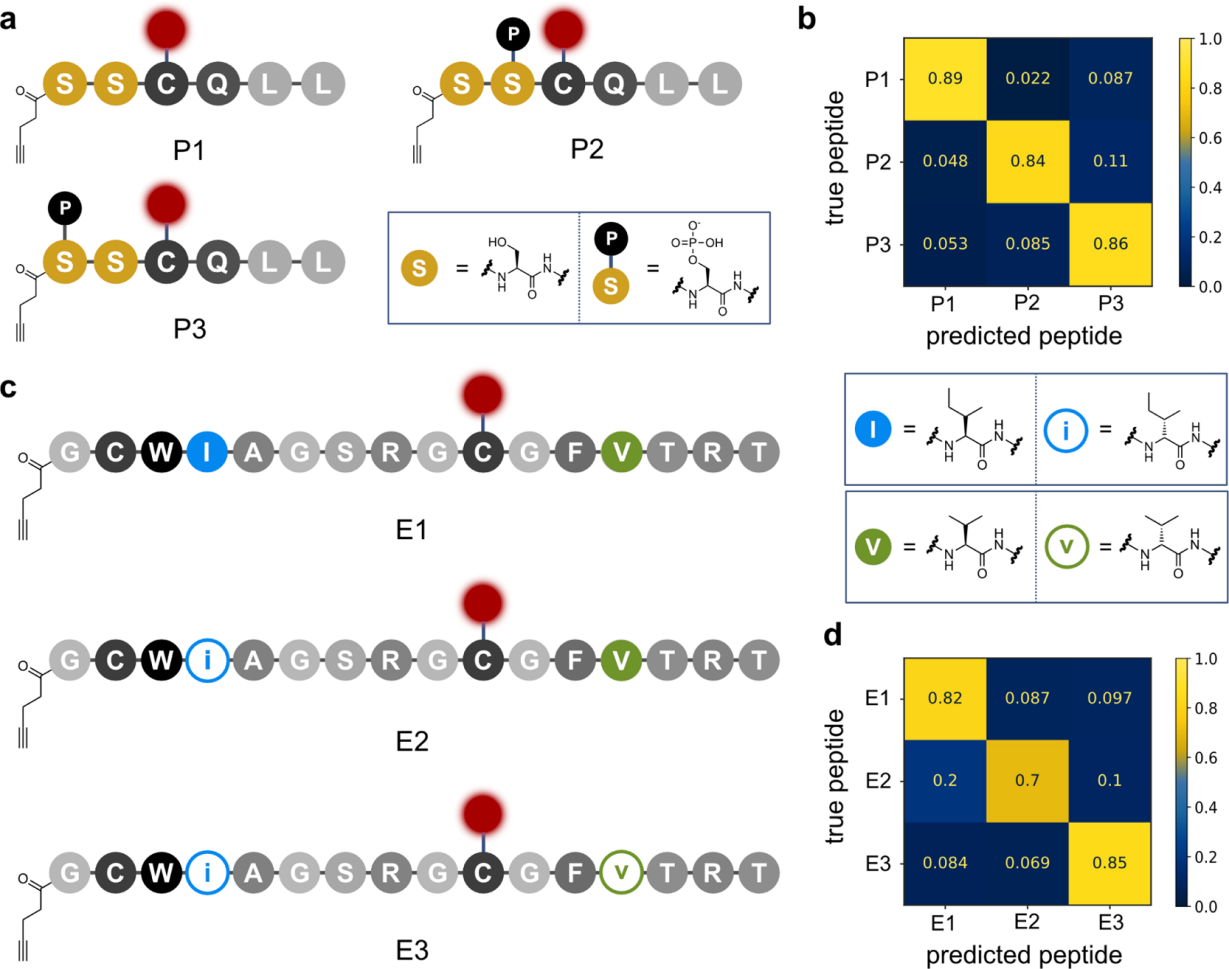
Single-molecule identification of peptide sets **P1–P3** and **E1–E3**. (a) Structures of peptides **P1–P3**. The chemical structures of serine (S) and phosphoserine are displayed in the box. (b) Confusion matrix displaying the results for the classification of 449 traces of peptides **P1–P3**. (c) Structures of peptides **E1–E3**. The chemical structures of l-isoleucine (I) and l-valine (V), as well as their epimers d-*allo*-isoleucine (i) and d-valine (v), are displayed in the box. (d) Confusion matrix displaying the results for the classification of 618 traces of peptides **E1–E3**.

Having proved that blinkognition can accurately distinguish between peptides with different sequences, we tested whether we could use it to detect the presence and position of PTMs. First, we studied the phosphorylation state of the guanosine triphosphate-binding protein Rap1B.^30^ Near the C terminus of Rap1B, serine residues S179 and S180 can be phosphorylated by cyclic adenosine monophosphate-dependent protein kinase A.^31^ These phosphorylation sites are part of a short peptide (SSCQLL) that could be obtained by proteolytic cleavage of the protein between residues K178 and S179. Thus, we synthesized the three relevant peptides (**P1–P3**, Figure 4a), and their blinking patterns were measured as described before. Using blinkognition, the phosphorylation state of peptides **P1–P3** could be determined with accuracies of >84% (Figure 4b). This result demonstrates that blinkognition is sensitive to both the presence and position of PTMs.

Next, we explored whether much more subtle PTMs, such as epimerization, could be detected by peptide blinkognition. For this purpose, we chose OspA, a ribosomally synthesized and post-translationally modified peptide (RiPP) of cyanobacterial origin (*Oscillatoria* sp. Pasteur Collection of Cyanobacteria (PCC) 6506).^32^ This peptide is consecutively post-translationally epimerized at isoleucine (I4) and valine (V13) residues by the *S*-adenosyl-l-methionine radical epimerase OspD to afford d-valine (v13) and d-*allo*-isoleucine (i4) residues.^33^ We prepared the parent, all-l, peptide and those containing either only i4 or both i4 and v13 (**E1–E3**, Figure 4c) and recorded their blinking patterns as described before. Even in this very challenging peptide fingerprinting case, we could obtain an overall classification accuracy of 79% (Figure 4d). Although enantiomers of isolated amino acids have been identified before by recognition tunneling^13^ or using single-molecule junctions,^34^ to the best of our knowledge, this is the first example of single-molecule identification of peptides solely differing by a single epimerized residue. Moreover, the fact that these hexadecapeptides (**E1–E3**) are classified with comparable accuracies to penta- and hexa-peptides (**C1–C4** and **P1–P3**, respectively) demonstrates that, unlike sequencing methods, the accuracy of blinkognition does not inherently decrease in longer peptides.

Blinkognition is suitable for hypothesis-driven studies in which specific peptides are targeted for quantification. In its present form, blinkognition is not capable of sequencing or identifying a peptide for which no training data exist. Furthermore, we have only demonstrated the identification of peptides in very simple mixtures. Nevertheless, several inherent advantages make blinkognition a unique single-molecule proteomics approach worthy of further development. It provides a fast and simple way to identify peptides, even when they differ from each other only by subtle PTMs. Extending our approach to the identification of other peptides or PTMs does not depend on trace prediction but rather requires the preparation of the corresponding pure standards for model training. Unlike other single-molecule fluorescence approaches,^14,15^ blinkognition does not rely on chemical degradation or proteolysis steps; thus, it is faster, simpler, and less prone to artifacts. Although other single-molecule fluorescence fingerprinting approaches exist,^19,20^ blinkognition has the advantage of providing information about the presence of PTMs on amino acids that have not been labeled. Compared to nanopore sequencing,^10^ blinkognition does not require denaturation of the peptide, can be applied to both negatively and positively charged peptides (e.g., **C1–C4** and **E1–E3**, respectively), and could be massively parallelized to produce millions of single-molecule reads, similarly to next-generation DNA sequencing.

Similar to other recent studies in this field,^10,14^ so far, we have relied on synthetic peptides that are functionalized for click chemistry. To be able to analyze naturally occurring peptides and proteins, strategies for surface immobilization and amino-acid-specific labeling have to be developed. These experiments are beyond the scope of this initial study, but recent advances in protein bioconjugation could be leveraged for both surface immobilization and installation of the spontaneously blinking dye. For example, a recent study has ranked several amino-acid-specific conjugation reactions based on their selectivity and coverage of the proteome.^35^ This study provides a good starting point to find suitable reactions to label specific amino acids within natural peptides and proteins with both spontaneously blinking dyes and surface anchors. We also envision the use of vesicle encapsulation and surface attachment of fluorescently labeled macromolecules for TIRF imaging.^36^ This encapsulation method would eliminate the need for alkyne functionalization and click conjugation of the peptide to the surface. Furthermore, we also envision that for longer peptides or proteins, more than one amino acid could be labeled with the blinking fluorophore. Having more than one dye per peptide could be an advantage since each fluorophore would report on its own region of the peptide, and the distance between the fluorophores would also be reflected in energy transfer and other inter-fluorophore interactions that may affect the blinking pattern. Since blinking patterns are not predicted ab initio, but rather measured experimentally and used to train a deep learning model, the combined blinking patterns of multiple fluorophores would contain more information about the peptide. This additional information could be used by the model to classify it with higher accuracy.

Finally, we envision that blinkognition could be applied to other macromolecules for which few analytical methods exist, such as oligosaccharides. Overall, blinkognition represents a new avenue for the development of single-molecule analytical technologies, with a potential impact on both basic research and clinical applications.

## Supporting Information Available

General experimental methods, details of peptide synthesis, preparation of glass slides, fluorescence imaging protocols, description of data analysis and ML pipelines, methods of small-molecule synthesis, single-molecule images on glass slides, randomly selected blinking traces, correlation plots, PCA, confusion matrices for classical machine and deep learning models, results of nested cross-validation, explanation of uncertainty prediction with Monte Carlo dropout, noise classification control experiments, certainty filtering control experiments, ^1^H and ^13^C of small molecules, and high-resolution LC–MS characterization of all peptides (PDF)

## Notes

Single-molecule time traces at different stages of analysis, selected Jupyter notebooks, and sample raw time-lapse acquisitions are available on Zenodo (DOI: 10.5281/zenodo.7414715). Full code for single-molecule fluorescence trace extraction, normalization, and deep learning classification of peptides is available on GitLab (https://gitlab.uzh.ch/locbp/public/blinkognition). Raw time-lapse acquisitions are available upon request.
