# Supplementary material for: Single-Molecule Peptide Identification Using Fluorescence Blinking Fingerprints
Source: J Am Chem Soc. 2023 Jan 5;145(2):1441–7. doi: 10.1021/jacs.2c12561 (PMC9853850; doi:10.1021/jacs.2c12561)
Supplement: Supplementary file 1 — ja2c12561_si_001.pdf [file ja2c12561_si_001.pdf]

Supplementary Information for

Single-Molecule Peptide Identification using Fluorescence Blinking Fingerprints

Salome Püntener[1,2] and Pablo Rivera-Fuentes[1,2]*

1) Institute of Chemical Sciences and Engineering, Ecole Polytechnique Fédéral de Lausanne, CH-1015, Lausanne, Switzerland.
2) Department of Chemistry, University of Zurich, CH-8057, Zurich, Switzerland.

*Correspondence to: pablo.riverafuentes@uzh.ch

1 Supporting Methods 2
General methods 2
Peptide synthesis 2
Preparation of glass slides 3
Fluorescence imaging 5
Data analysis 6
2 Small-Molecule Synthesis 9
3 Supporting Figures 13
4 Supporting Tables 44
5 References 48

1 Supporting Methods

General methods

All reagents were purchased from commercial sources and used as received. Anhydrous solvents were procured from Acros Organics and used as received. All solvents used in the preparation of the coverslips were HPLC grade. NMR spectra were acquired on Bruker AVANCE NEO-400, Bruker AVANCE III-400, Bruker AVANCE III HD-600, or Bruker AVANCE II-800 instruments. ^{1}H NMR chemical shifts are reported in ppm relative to $SiMe_4$ (δ = 0) and were referenced internally with respect to residual protons in the solvent (δ = 7.26 for chloroform, δ = 1.94 for CH_3CN, δ = 3.31 for CH_3OH and δ = 3.58 for THF). Coupling constants are reported in Hz. ^{13}C NMR chemical shifts are reported in ppm relative to $SiMe_4$ (δ = 0) and were referenced internally with respect to solvent signal (δ = 77.16 for $CDCl_3$ and δ = 1.32 for CD_3CN). Preliminary peak assignments are based on calculated chemical shifts and multiplicity. Low-resolution mass spectra (LRMS) were acquired on a Shimadzu LC-MS 2020 spectrometer by using electrospray ionization (ESI). Purification by flash column chromatography and prep-HPLC was performed using a Büchi Pure-Chromatography-System and Büchi FlashPure columns. All the code was written in Python and the deep learning model was implemented using TensorFlow 2 and Keras.[1,2] All confusion matrices were plotted using Scikit-learn with matplotlib integrated.[3,4] The statistical plots were generated using the Seaborn package with the included statistical methods.[5] The synthesis of small molecules **S1**-**S6** is described in the next section and their spectroscopic data are displayed in Figures S17-S29.

Peptide synthesis

General information: Peptides **C1**-**C4** and **P1**-**P3** were prepared by manual SPPS, whereas peptides **E1**-**E3** were prepared on an automated Tribute™ UV-IR peptide synthesizer (Gyros Protein Technologies AB) with manual coupling of the HMSiR labeled cysteine at an intermediate step. All peptides were synthesized on a 0.05 mmol scale.

Manual peptides synthesis: After every reaction and swelling step, the resin was washed three times with each CH_2Cl_2 and DMF (4 mL). The swelling and all the reaction steps were shaken at room temperature using a heating/cooling dry bath (Thermo Scientific). Peptides were synthesized on 100 mg pre-loaded Wang-resins (Bachem, 0.5 – 0.8 mmol g^{-1}, 1 equiv.), which were swollen for at least 3 h in 4:1 CH_2Cl_2/ DMF. Standard, manual Fmoc-SPPS protocols were used for synthesis. Briefly, deprotection was performed with 20% piperidine in DMF for 20 min and coupling with *O*-(1H-Benzotriazole-1-yl)-*N*,*N*,*N*',*N*'-tetramethyluroniumhexafluorophosphate (HBTU) (4 equiv.), DIPEA (8 equiv.)

and Fmoc-Xaa-OH (4 equiv.) in DMF for 1 h. Fmoc-Cys(HMSiR)-OH **S5** was coupled with 1-hydroxbenzotriazole (HOBt) (2.5 equiv.), N-Ethyl-N′-(3-dimethylaminopropyl) carbodiimide hydrochloride (EDC HCl) (2.5 equiv.), Fmoc-Cys(HMSiR)-OH **S5** (2.5 equiv.). The solids for Fmoc-Cys(HMSiR)-OH **S5** coupling were pre-mixed in 3 mL DMF: THF 1:1 for 5 min, added to the deprotected resin and left shaking for 3 h. For the coupling phosphorylated amino acid **S6** the equivalents of the amino acid **S6**, coupling reagent, HBTU, and DIPEA were increased by one to five and nine equivalents respectively. The final coupling was carried out by pre-mixing pentynoic acid (10 equiv.) with EDC HCl (10 equiv.) in 2 mL of 1:1 CH_2Cl_2/ DMF after 2 min the mixture was added to the resin for 2 h. Cleavage from the resin and concurrent global deprotection was carried out with a mixture of trifluoroacetic acid (TFA), water and triisopropylsilane (TIPS) (90:5:5) for 3 h. The crude products were purified by preparative HPLC (A: H_2O + 0.1% TFA, B: CH_3CN; 1. 0-10 min, 20%-50% B; 2. 10-20 min, 50% B; 3. 20-24 min, 50-20% B). The obtained peptides were characterized by LC-MS, ESI-HRMS and ESI-MS/MS (Figures S30-S39). Pure peptides were stored as 1 mM stock solutions in DMSO or in lyophilized form.

Automated peptides synthesis: Peptides **E1**-**E3** were synthesized on a Tribute UV-IR (Gyros Protein Technologies) peptide synthesizer applying the manufacturer's standard cycles. The couplings were conducted with Fmoc-Xaa-OH (5 equiv.), O-(1H-6-chlorobenzotriazole-1-yl)-1,1,3,3-tetramethyluronium (HCTU) (4.75 equiv.) and DIPEA (7 equiv.) in DMF. The Fmoc-deprotection was done with 20% piperidine in DMF. The coupling of the Fmoc-Cys(HMSiR)-OH **S5** amino acid was conducted manually with Fmoc-Cys(HMSiR)-OH **S5** (2.5 equiv.), HOBt (2.5 equiv.), EDC HCl (2.5 equiv.). The solids were premixed in 3 mL DMF: THF 1:1 for 5 min, added it to the deprotected resin and left shaking for 3 h. The final coupling was done manually by pre-mixing pentynoic acid (10 equiv.) with EDC HCl (10 equiv.) in 2 mL of 1:1 CH_2Cl_2/ DMF after 2 min the mixture was added to the resin for 2 h. After the manual steps the resin was washed with each CH_2Cl_2 and DMF (4 mL). The peptides were cleaved from the resin by addition of 3 mL of Reagent K ((TFA, phenol, water, thioanisole, 1,2-ethanedithiol in ratio; 82.5:5:5:5:2.5) mixing for 3 h. After concentration of the cleavage mixture cooled *tert*-butyl methyl ether (MTBE) was added to the residue. The precipitate was centrifuged for 15 min at 4000 rpm, the supernatant was removed, the pellet was resuspended in MTBE and centrifuged once more. The crude products were purified by HPLC. The obtained peptides were characterized by ESI-HRMS and ESI-MS/MS. They were stored as 1 mM stock solutions in DMSO or in lyophilized form.

Preparation of glass slides

The fluorophore-peptide conjugates were covalently bound to glass slides that were extensively cleaned, followed by passivation using a mixture of heterobifunctional polyethylene glycol derivatives (PEGs), adapted from

published procedures.[6] For passivation a mixture of PEGs that are non-reactive (methoxy-PEG_{5000}-NHS) and reactive (azide-PEG_{5000}-NHS) in a "click" reaction. This mixture allowed for sparse single-molecule signals on the glass surface.

Surface passivation: The glass containers and Erlenmeyer dedicated to the aminosilation step were sonicated first with 1 M KOH, rinsed with ultrapure water five times, sonicated for 20 min in CH_3OH and dried under a stream of nitrogen. The coverslips (always in pairs) were placed into the cleaning containers and rinsed with ultrapure water. A 10% Alconox solution was added and the slides were sonicated for 20 minutes. The slides were rinsed with tap water until no bubbles were apparent, three times with distilled water, twice with ultrapure water and sonicated for 5 min in ultrapure water. The container was rinsed with acetone once and refilled with acetone and sonicated for 20 min. After the last rinse with acetone, the coverslips were dried under a stream of N_2 and placed in the active area of a UV/ozone cleaner (Jelight Company Inc., UVO-Cleaner Model No. 256-220) and treated for 10 minutes followed by 5 min of resting time. Results of the different cleaning steps are displayed in Figures S2-S3. The clean and activated coverslips were transferred into the containers dedicated to the aminosilation step. The aminosilation solution was freshly prepared in the cleaned Erlenmeyer by adding 2 mL of *N*-(2-aminoethyl)-3-aminopropyltriethoxysilane (AEAPTES)- to 98 mL of acetone (HPLC grade) and mixing with a dedicated glass rod. The pre-mixed solution was added to the slides, and they were placed in a dark, well-leveled place. After 10 min the containers were sonicated for 1 min and then put back in the dark for another 10 min. The aminosilation solution was discarded and the slides were rinsed with acetone once and three times with ultrapure water before drying them with N_2 and placing them in the PEGylation container (a pipette tip box with tips on the edges of the coverslips, to prevent them from touching the box with the clean surface).

For PEGylation the buffer and the solution were freshly prepared right before application. For five pairs of coverslips, 1 mg azide-PEG5000-NHS and 16 mg methoxy-PEG5000-NHS were added to an Eppendorf tube and dissolved in 320 μL PEGylation buffer (1 mM sodium bicarbonate solution). This solution was mixed gently and centrifuged for 1 min at 10 000 rpm. 70 μL of PEGylation solution was added onto the functionalized side of half of the coverslips, then a second coverslip was gently placed face down on top of the solution without introducing any bubbles. Water was added in the bottom of the pipette box and the slides were kept closed in a dark, well-leveled place for 3 h. The coverslips were taken apart, rinsed with ultrapure water and CH_3OH from a squirt bottle and dried with compressed nitrogen.

For the second PEGylation step, 7 μL of a 250 mM MSPEG4 solution in anhydrous DMF was added to 63 μL of PEG buffered (prepared before) and added in the same manner to a pair of coverslips. The slide "sandwiches" were incubated overnight. The slides were taken apart and rinsed with CH_3OH and ultrapure water, and dried under a stream of N_2.

Click functionalization standard procedure: The coverslips were placed back into the pipette tip boxes with the passivated surface face up. For the measurement of three different peptides in one experiment, a master-mix with the common components was prepared: 45 μL of a 100 mM tris-hydroxypropyltriazolylmethylamine (THPTA, 10 mM, final concentration) were combined with 9 μL of a 100 mM copper (II) sulfate solution (2 mM, final concentration). The reagents were diluted with 351 μL of a 3:1 water/glycerol mixture.

From this diluted solution, 135 μL were transferred to a new Eppendorf tube, then 0.15 μL of the corresponding 1 μM peptide-dye conjugate stock solution was added, followed by 15 μL of 1 M sodium ascorbate. The reaction mix was vortexed thoroughly before applying 70 μL of the final reaction solution to the reactive surface of a passivated glass slide. A second glass coverslip is gently placed on top, with the passivated side facing down, i.e., towards the reaction solution, without introducing any bubbles. After 2 h the slides were slid apart and rinsed with ultrapure water, 25 mM enediaminetetraacetic acid (EDTA) solution, ultrapure water, acetone, EtOAc, CH_3OH, and finally again with ultrapure water. The functionalized coverslips were dried under a stream of $N_{2,}$ placed on a KimWipe^{TM}-lined container and transported to the microscope for imaging.

Fluorescence imaging

Single-molecule movies were acquired on a Nikon N-STORM microscope (Nikon, UK Ltd.) with an SR Apochromat TIRF 100x 1.49 N. A. oil immersion objective lens. A piezo-electronic focus-lock system (perfect focus system) was used to prevent axial drift during data acquisition. The illumination power of the diode-pumped solid-state laser was measured at the tip of the optical fiber as 638 nm, 110 mW. The emission was passed through a filter with a band pass window at 683-783 nm. The fluorescence was detected with an EMCCD camera Andor iXon Ultra 888. The microscope was operated using the NIS Elements (Nikon) software. For imaging, the slides were fixed in a cell chamber and rinsed twice with 1 mL of imaging buffer (10 mM sodium phosphate buffer at pH = 7.4), 1 mL of imaging buffer was added for imaging. The coverslip was then imaged in TIRF mode with a 638 nm laser with an exposure time of 30 ms. The conditions were kept constant for all acquisitions. A field of view was imaged for 6000 frames. For all peptides, single-molecule measurements were

carried out on three different days and at least two different coverslips per day, and all these signals were mixed to create the dataset for further analysis.

Data analysis

To perform the classification analysis, we first localized the single-molecule signals, extracted the numeric intensity values of the signal over all frames followed by data normalization, filtering to remove noise signals as described below. For deep learning, the standardized and normalized traces were used directly as input for the model. All deterministic ML analysis was conducted on a desktop computer with an AMD Ryzen 9 3900X 12-Core, 3800 MHz Processor, a GeForce RTX 2070 Super graphics card and 128 GB of physical memory. The deep learning model was trained using the GPU. The MCD models were trained on the ScienceCluster (Tesla V100-SXM2-32GB GPU) and evaluation and analysis on a ScienceCloud virtual machine instance using 8 cores of an AMD EPYV 7702 Hypervisor CPU and an nVidia Tesla T4 GPU, service infrastructure provided by S3IT (www.s3it.uzh.ch), the Service and Support for Science IT team at the University of Zurich.

Fluorescence trace extraction: In each frame all the single molecules were localized using the freely available single-molecule localization package Picasso[7] with the settings listed in Table S1. The obtained framewise localizations were combined using the Picasso postprocess module. All the localizations within a two-pixel distance from the first appearance of a localization were combined as one particle and the final location of the particle was calculated from the averaged x- and y-coordination of the combined localization that were detected. No significant lateral drift was detected during the acquisition. The mean locations were exported for all the particles and further used to obtain the fluorescence intensity trace of each particle with custom-written code. A mean location was used to define a box with a side length of five pixels and the mean as its center. The pixel intensities within this box were summed up and recorded as the particle intensity for the respective frame, resulting in an intensity trace over all the frames. All boxes that overlapped in more than two pixels were not considered, as well as boxes that overlapped with the edge of the field of view. All traces were recorded and saved in reference to their peptide of origin (**C1**-**C4**, **P1**-**P3**, or **E1**-**E3**).

Fluorescence trace preprocessing and filtering: Traces obtained from the movies needed to be standardized to remove potential influences from the different technical replicates (different coverslips and different days). Therefore, we used the last 500 frames, which were bleached in most traces, to estimate the mean value of the background of the acquisition. This value was subtracted from each frame in the trace. Furthermore, we standardized the fluorescence intensity values by either calculating the z-score for each value in the traces or by producing the min-max normalized traces. The z-scored traces were used

to determine the peaks in each trace using the function find_peaks, contained in the SciPy python library.[8] As a threshold for peak detection, we set a minimum intensity of eight standard deviations (8σ) from the background. These peaks were the basis for the first step of trace filtering to remove spurious traces stemming from background noise or impurities in the experiment. The applied filtering criteria are listed in Table S2.

Feature determination, visualization, and classical ML: The z-scored traces were used to determine the peaks in each trace using the function find_peaks, contained in the SciPy python library.[8] Peak-based features were calculated from the found peaks and their properties. The peak width corresponds to the ON-time of the fluorophore, while the time between peaks corresponds to the OFF-time of the peaks. The total blinking time corresponds to the time from the left bounds of the first peak to the right bounds of the last peak The approximate signal area is the product of the peak height and the peak width. For these aspects of each trace and its peaks, we determine the maximum, minimum, mean, and standard deviation value.[9] Furthermore, we used the librosa package to calculate the tempo of the trace, when interpreting the signal trace as a musical onset envelop, which exhibited a low variation in all the traces.[10] The Fourier transformation of some traces was calculated for visual inspection but not used further for feature engineering. For visualization and inspection of the features, correlation plots were used with a diagonal corresponding to density-normalized distribution plots. The principal component analysis (PCA) approach implemented in scikit-learn was applied using the standard normalized features. For classical machine learning (ML) models, the features were normalized using RobustScaler in scikit-learn.[3] The normalized features were used to train a selection of classical ML models using a grid search with 5-fold cross-validation using a seed 13102022.[3]

Data augmentation for deep learning: To increase the amount of data for improved learning of model training, we generated traces with a mirror-image peak region. We defined the peak region between the left-bound of the first and the right-bound of the last detected peak. This partial sequence was inverted in place, to obtain the trace with a mirrored peak-region.

Deep-learning settings using a one-dimensional convolutional neural network (1D-CNN) classification using a deterministic approach (1D-CNN-GRU): The deep learning model was implemented using Python and TensorFlow 2.7 [2] with the Keras API [1]. The hyperparameters used in the model are described in Table S7. We used Adam[11] optimizer, with default settings, with a mini-batch size of 32 for the compounds peptides set **C1**-**C4** and **P1**-**P3** and a size of 128 for the set **E1**-**E3**. The traces obtained after preprocessing, filtering, and data augmentation were used for the deep learning approach. The z-scored and min-max scaled traces were used as separate features for each

trace and directly used as input vectors to the model. To prevent overfitting and stop the training at the best performance on the validation set, we used early stopping with a patience of 10 epochs. For a more general sense of model behavior and to avoid potential outlier results, the model was trained using a nested cross-validation approach (Figure S11). We report the mean value of all the folds in the confusion matrices and the variation of the true positive rate along with the mean overall accuracies and standard deviations of the model evaluation on a separate test set that was not used in model training or validation (Table S8). We performed all the modeling experiments using a seed of 42 unless mentioned otherwise.

Monte Carlo dropout (MCD) implementation (1D-CNN-GRU-MCD): The traces were preprocessed as in the deterministic approach and the same input was used for the probabilistic model 1D-CNN-GRU-MCD (Table S9). To make the model probabilistic, custom Dropout and GRU layers were used, which use MCD at inference time. As MCD was used in the GRU layer itself, the input Dropout layer was removed. For evaluation, 100 predictions were calculated for each trace in the validation/test set yielding 100 potentially different predictions depending on the dropped nodes by the dropout mechanism. The mean model output over the 100 predictions was calculated per class used to predict the label.

Definition of the classification certainty threshold (CT) for trace retention: The uncertainty quantification was calculated as the Wasserstein distance (using the SciPy implementation)[8] between the class with the highest mean probability over 100 predictions and the closest other class in the classification problem. Therefore, the prediction with the highest mean was determined and the Wasserstein distance for the other two or three classes in the problem were calculated, the minimal distance was determined and stored (Figure S12). A CT value was chosen ($0 \leq CT \leq 1$) and used to filter uncertain traces if their minimal distance was larger than the chosen CT (CT = 0.7 for all cases in this work). All traces that were not filtered out based on the CT were used to calculate the accuracy score.[3] Importantly, the CT value does not depend on whether the class with the highest probability is correct. Larger CT values led to higher classification accuracies, but also to a larger loss of traces (Figure S14). This increase in accuracy, however, is not driven by simply having a smaller test set, since discarding traces at random did not lead to higher accuracy (Figure S15). Finally, up to CT = 0.7, we did not observe significant changes in the relative number of traces per peptide class in the test sets (Figure S16).

2 Small-Molecule Synthesis

A general synthetic scheme is provided in Figure S1.

3,3'-(Dimethylsilanediyl)bis(*N,N*-dimethylaniline) (S1)

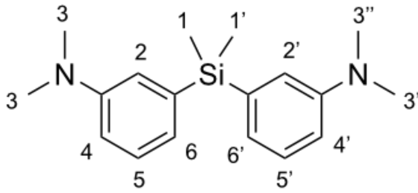

3-Bromo-*N*,*N*-dimethylaniline (5 g, 25 mmol) was dissolved in dry THF (60 ml) and the solution was cooled to –78 °C. *n*-Butyllithium (1.6 M, 17.2 mL, 27.5 mmol) was added dropwise and the mixture was stirred at –78 °C for 2 h. Dichlorodimethylsilane (1.52 mL, 12.5 mmol) was added dropwise and the mixture was stirred for 2 h at 25 °C. Brine (40 mL) and H_2O (10 mL) were added and the mixture was extracted three times with EtOAc. The combined organic phases were dried over $MgSO_4$ and concentrated onto Celite. The crude was purified by flash column chromatography (SiO_2; hexane to hexane/EtOAc 9:1) to give the product as a light-yellow oil (3.6 g, 50%).

^{1}H NMR (400 MHz, $CDCl_3$) δ = 7.31–7.19 (m, 2H, H5), 6.97–6.90 (m, 4H, H4, H2), 6.78 (dd, *J* = 8.3, 2.8, 2H, H6), 2.94 (s, 12H, H3), 0.55 (s, 6H, H1) ppm.

^{13}C NMR (101 MHz, $CDCl_3$) δ = 154.75, 141.11, 131.49, 125.30, 120.50, 116.20, 42.84, 0.84 ppm.

3,3'-(Dimethylsilanediyl)bis(4-bromo-*N,N*-dimethylaniline) (S2)

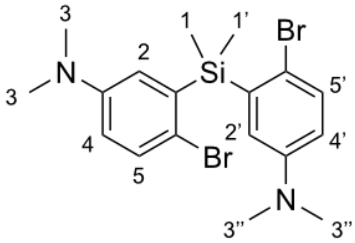

3,3'-(Dimethylsilanediyl)bis(*N*,*N*-dimethylaniline **S1** (3.00 g, 0.01 mol) was dissolved in CH_3CN (75 mL) in a flame-dried flask. The solution was cooled to 0 °C and *N*-bromosuccinimide (3.75 g, 0.021 mmol) was added in small portions. After complete addition, the solution was stirred for 1.5 h at 25 °C then sat. $NaHCO_3$ was added to neutralize the solution. The organic phase was washed with water. The combined aqueous phases were extracted twice with CH_2Cl_2. All CH_2Cl_2 fractions were combined dried over $MgSO_4$ and concentrated under reduced pressure. The residue was purified by flash column chromatography. (SiO_2; hexane: EtOAc 95:2) yielding a white product (2.69 g, 59%).

^{1}H NMR (400 MHz, $CDCl_3$) δ = 7.36 (d, *J* = 8.8, 2H, H5), 6.85 (d, *J* = 3.2, 2H H2), 6.61 (dd, *J* = 8.7, 3.2, 2H, H4), 2.89 (s, 12H, H3), 0.77 (s, 6H, H1) ppm.

^{13}C NMR (101 MHz, $CDCl_3$) δ = 149.02, 138.86, 133.10, 121.92, 116.94, 115.40, 40.70, 27.05, -0.51, -0.78, -1.06 ppm.

5'-Bromo-*N*3,*N*3,*N*7,*N*7,5,5-hexamethyl-3'*H*,5*H*-spiro[dibenzo[*b*,*e*]siline-10,1'-isobenzofuran]-3,7-diamine (S3)

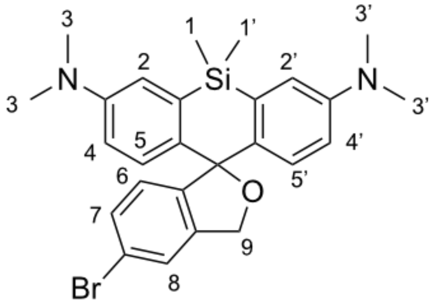

Compound **S2** (1 g, 2.19 mmol) in dry THF (10 mL) was added to magnesium turnings (213 mg, 8.77 mmol) in dry THF (3 mL) at 60 °C followed by addition of 1,2-dibromoethane (40.6 µL, 470 µmol). The mixture was stirred at 25 °C for 3 h and the solution slowly turned yellow. The Grignard reagent was transferred into an empty flame-dried Schlenk flask and a solution of 5-bromophthalide (170 mg, 783 µmol) in dry THF (8 mL) was added at 60 °C. Upon addition, the reaction turned from deep yellow to dark brown. The mixture was stirred at the same temperature for 12 h. Aqueous HBr (3.8 mL, 47%) was added and the solution turned orange red and CH_2Cl_2 was added. $NaHCO_3$ was added very carefully until the color moved to the CH_2Cl_2 phase. The aqueous phase was extracted three more times with CH_2Cl_2. The organic phase was dried over Na_2SO_4, concentrated under reduced pressure and loaded onto Celite. Purification by reverse phase column chromatography (SiO_2-C_{18}; CH_3CN / H_2O + 0.1 TFA 5:95 to CH_3CN / H_2O + 0.1 TFA 95:5) gave the compound **S3** as the blue trifluoroacetate salt (302 mg, 45%).

^{1}H NMR (400 MHz, MeOD) δ = 7.90 (s, 1H, H8), 7.63 (d, *J* = 8.0, 1H, H7), 7.35 (d, *J* = 2.8, 2H, H2), 7.11–7.04 (m, 3H, H5, H6), 6.79 (dd, *J* = 9.7, 2.9, 2H, H4), 4.28 (s, 2H, H9), 3.35 (s, 12H, H3), 0.60 (d, *J* = 2.6, 6H, H1) ppm.

^{13}C NMR (101 MHz, MeOD) δ = 167.63, 159.17, 158.76, 155.81, 149.41, 143.39, 142.14, 137.21, 132.07, 131.11, 131.05, 128.35, 124.14, 122.30, 120.28, 117.46, 115.33, 114.63, 111.80, 66.97, 61.74, 40.94, 27.20, -1.10, -1.34 ppm.

HRMS (ESI/QTOF) calcd. for $[C_{26}H_{30}BrN_2OSi]^+$: 493.1305; found 493.1305.

5'-Iodo-*N*3,*N*3,*N*7,*N*7,5,5-hexamethyl-3'*H*,5*H*-spiro[dibenzo[*b*,*e*]siline-10,1'-isobenzofuran]-3,7-diamine (S4)

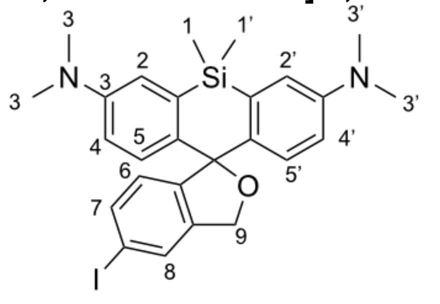

A flame dried microwave vial was charged with compound **S3** (49.5 mg, 100 µmol), CuI (9.52 mg, 50 µmol), NaI (300 mg, 2.00 mmol), and briefly evacuated and backfilled with N_2. Racemic *trans*-(1R,2R)-*N*,*N*'-dimethyl-cyclohexane-1,2-diamine (1.42 mg, 10 µmol), and dioxane (0.5 mL) were added. The microwave vial was sealed and the mixture was stirred at 110 °C for 29 h. The resulting suspension was cooled to reach 25 °C, diluted with 25% aq. NH_3, poured into water, and extracted with CH_2Cl_2. The combined organic phases were dried over Na_2SO_4 and concentrated under reduced pressure. The residue was purified by flash chromatography (SiO_2; hexane + 1% NH_3/ EtOAc + 1% NH_3 to 9:1 hexane + 1% NH_3 EtOAc + 1% NH_3) to provide product **S4** as a white solid (190 mg, 77%).

^{1}H NMR (400 MHz, $CDCl_3$) δ = 7.64 (s, 1H, H8), 7.53 (d, *J* = 8.0, 1H, H7), 7.24–7.14 (m, 2H, H2), 7.03 (d, *J* = 8.8, 2H, H5), 6.83 (dt, *J* = 8.8, 5.2, 2H, H4), 6.77 (s, 1H, H6), 5.08 (s, 2H, H9), 2.98 (s, 12H, H3), 0.56 (s, 3H, H1 or H1'), 0.49 (s, 3H, H1 or H1') ppm.
^{13}C NMR (101 MHz, ($CDCl_3$) δ = 141.12, 136.61, 120.08, 118.10, 116.43, 114.68, 92.21, 0.38, -0.99 ppm.
HRMS (ESI/QTOF) calcd. for $[C_{26}H_{30}IN_2OSi]^+$: 541.1167; found 541.1167.

_N_-(((9_H_-Fluoren-9-yl)methoxy)carbonyl)-_S_-(3,7-bis(dimethylamino)-5,5-dimethyl-3'_H_,5_H_-spiro[dibenzo[_b_,_e_]siline-10,1'-isobenzofuran]-5'-yl)-_L_-cysteine (S5)

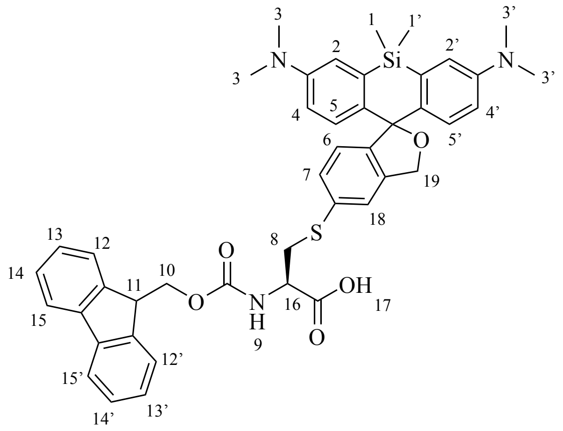

Xanthphos Pd G3 (15.8 mg, 16.7 µmol), Fmoc-L-Cys-OH (105 mg, 305 µmol) and compound **S4** (150 mg, 278 µmol) were dissolved in THF (1.4 mL) in a flame-dried Schlenk flask. The mixture was stirred at 25 °C for 2-3 min, then NEt_3 (65.6 µL, 472 µmol) was added, the Schlenk tube was capped with a rubber septum, evacuated and backfilled with argon. The solution was stirred at 25 °C for 30 min. The solvent was removed under reduced pressure. The residue was loaded onto Celite and purified by flash chromatography (SiO_2; CH_2Cl_2 to CH_2Cl_2/CH_3OH 95:5) to yield a blue/green powder (200 mg, 95%).
^{1}H NMR (400 MHz, THF) δ = 7.77 (d, *J* = 7.5, 2H, H15), 7.66 (t, *J* = 8.2, 2H, H12), 7.40 (s, 1H, H18), 7.34 (t, *J* = 7.4, 2H, H14), 7.25 (dd, *J* = 8.7, 5.4, 3H, H7 and H12), 7.03 (dd, *J* = 8.7, 3.0, 3H, H5 and H9), 6.96 (t, *J* = 3.1, 2H, H2), 6.86 (dd, *J* = 8.0, 4.4, 1H, H6), 6.64–6.55 (m, 2H, H4), 5.21 (s, 2H, H19), 4.49 (td, *J* = 8.0, 4.6, 1H, H16), 4.28 (dd, *J* = 7.3, 2.1, 1H, H10), 4.23 (s, 1H, H11), 3.49 (dd, *J* = 13.5, 4.8, 1H, H8), 3.25 (dd, *J* = 13.5, 7.9, 1H, H8'), 2.89 (d, *J* = 4.3, 12H, H3), 0.58 (s, 3H, H1), 0.46 (s, 3H, H1') ppm.
^{13}C NMR (101 MHz, THF) δ = 172.37, 160.18, 159.90, 157.01, 149.88, 148.42, 145.40, 145.37, 142.39, 135.89, 129.70, 128.49, 127.93, 126.26, 126.24, 125.18, 123.93, 120.76, 118.43, 115.44, 74.24, 55.05, 54.84, 54.74, 48.38, 46.81, 42.03, 38.27, 37.32, 0.51, -0.64 ppm.
HRMS (ESI/QTOF) calcd. for $[C_{44}H_{46}N_3O_5SSi]^+$: 756.2922; found 756.2913.

***N*-(((9*H*-fluoren-9-yl)methoxy)carbonyl)-*O*-((benzyloxy)(hydroxy)phosphoryl)-*L*-serine (S6)**

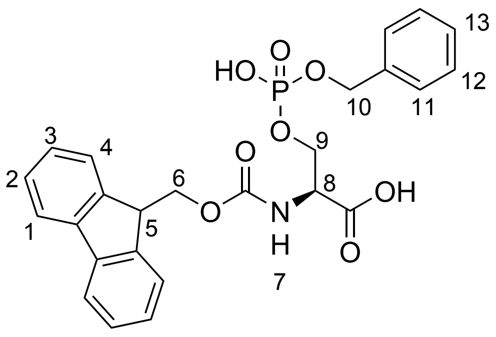

Water from Fmoc-*L*-Ser-OH·H_2O was azeotropically distilled from THF (4 mL g^{-1}) on the rotary evaporator and dried overnight. PCl_3 (379 µL, 4.34 mmol) was dissolved in THF (8.0 mL) and cooled to 0 ºC. Benzyl alcohol (518 µL, 5 mmol) was added while keeping the internal temperature below 5 ºC. The solution was stirred for 10 minutes at 0-5 ºC. The reaction was further cooled to –5 °C then 2,6-lutidine (1.17 mL, 10 mmol) was added to the flask keeping the reaction at –5 to 5 ºC, forming a thick slurry. 2,6-Lutidine (389 µL, 3.34 mmol) was added to a solution of Fmoc-Ser-OH (1.15 g, 3.34 mmol) in THF (4.0 mL) in a separate flask. This solution was added to the reaction at a rate that kept the mixture at –5 to 5 ºC. Upon reaction completion, H_2O (3.6 mL) was added to the flask, maintaining the temperature below 10 ºC followed by addition of NaBr (789 mg, 7.67 mmol) at 0 ºC. A 20% w/w aqueous solution of $NaBrO_3$ (252 mg, 1.67 mmol) was added at 0-5 ºC. After the addition, the mixture was warmed to 25 °C. Upon reaction completion, an aqueous solution of $Na_2S_2O_5$ (20% in H_2O) (1.0 mL) was added to the flask in one portion. 2-CH_3-THF was added, and the layers were shaken and separated. The organic layer was washed with brine, dried with Na_2SO_4, and concentrated under reduced pressure. The crude oil was diluted with 2-CH_3-THF (7 mL g^{-1}), stirring at ambient temperature for 16 h, yielded a white precipitate, which was filtered of and washed with cold 2-CH_3-THF. The product was obtained as a white solid (700 mg, 42%).

^1H NMR (400 MHz, DMSO-d_6) δ = 7.89 (d, *J* = 7.6 Hz, 2H, H1), 7.84 (d, *J* = 8.3 Hz, 1H, H7), 7.73 (d, *J* = 7.5 Hz, 2H, H4), 7.45–7.27 (m, 9H, H2, H3, H11, H12 and H13), 4.93 (d, *J* = 7.1 Hz, 2H, H10), 4.36–4.08 (m, 6H, H5, H6, H8, H9).

^{13}C NMR (101 MHz, DMSO-d_6) δ = 170.75, 155.98, 143.76, 143.74, 140.68, 136.77, 136.69, 128.36, 128.03, 127.64, 127.60, 127.08, 125.30, 120.10, 67.52, 67.47, 65.92, 65.28, 65.23, 54.40, 54.32, 46.55. ^{31}P NMR (162 MHz, DMSO) δ = -1.49.

HRMS (ESI): *m/z* calcd. $[C_{25}H_{24}NO_8P]^+$: 498.1312; Found 498.1327.

3 Supporting Figures

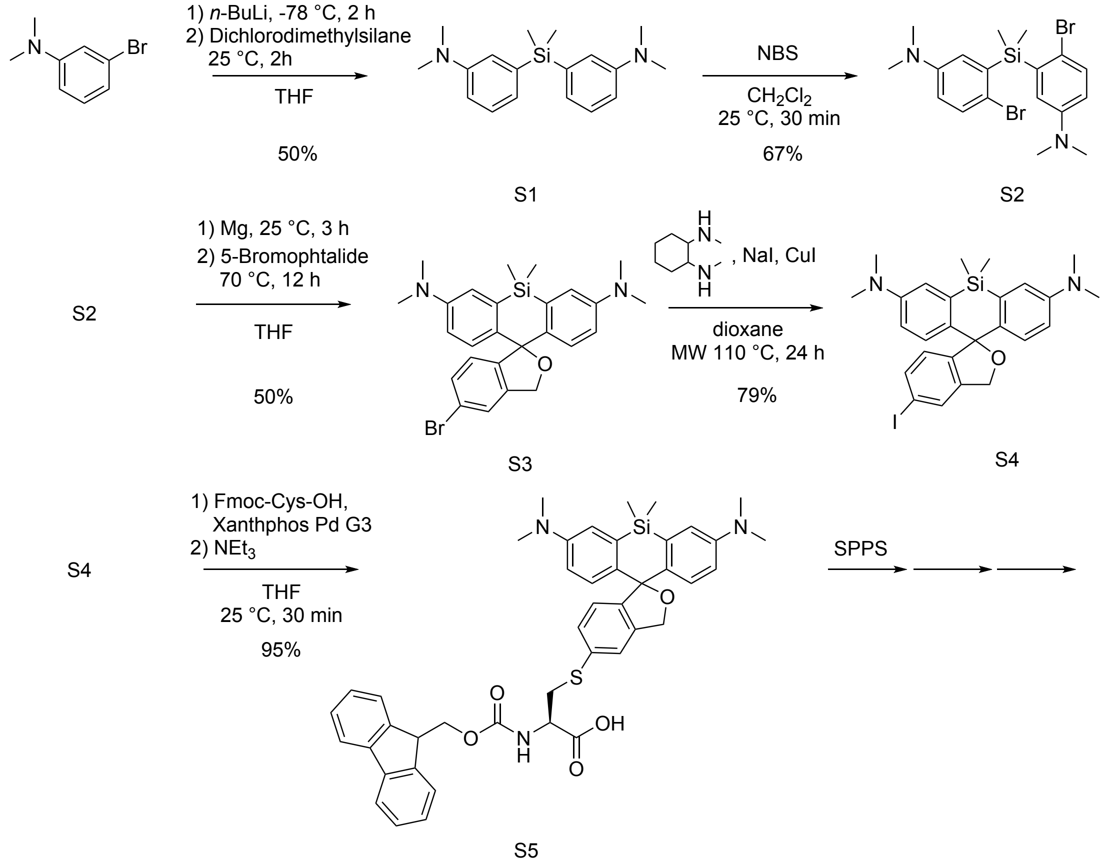

Figure S1. General overview of the synthesis of the HMSiR-Fmoc-cysteine building block **S5** that was used in SPPS to synthesize the model peptides with site-specific labelling.

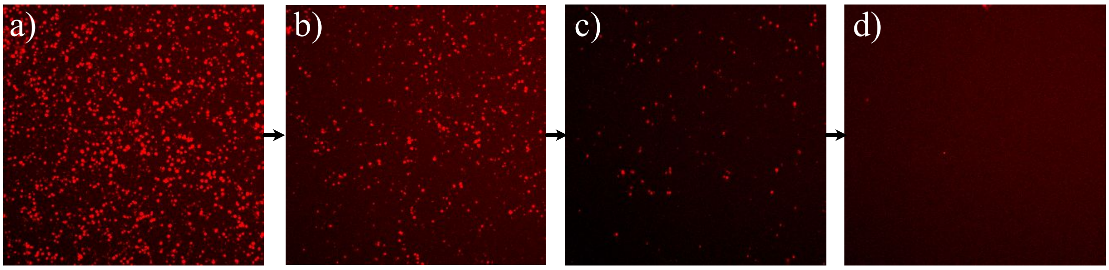

Figure S2. Images of glass slides at every step of the cleaning procedure. **a**, Commercial glass, out-of-the-box. **b**, Result after sonication in 10% Alconox™ solution for 20 min. **c**, Result after sonication in acetone for 20 min. **d**, Result after UV/ozone cleaning for 10 min with 5 min resting time. All images were obtained as the maximum projection over 6000 frames from slides of the same batch.

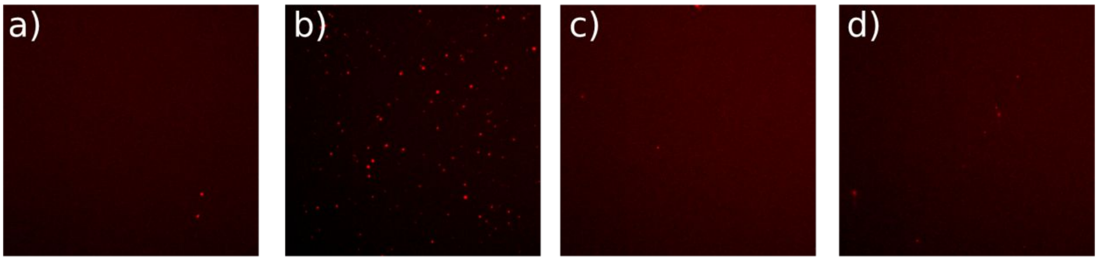

Figure S3. Comparison of final cleaning step procedures. **a**, Result after sonication in KOH for 40 min. **b**, Result after KOH treatment with an additional wash in freshly prepared Piranha solution for 20 min. **c**, Result after cleaning in a plasma cleaner using air plasma for 5 min. **d**, Result after UV/ozone cleaning for 10 min with 5 min resting time All images were obtained as the maximum projection over frames from slides of the same batch. time. All images were obtained as the maximum projection over 6000 frames from slides of the same batch.

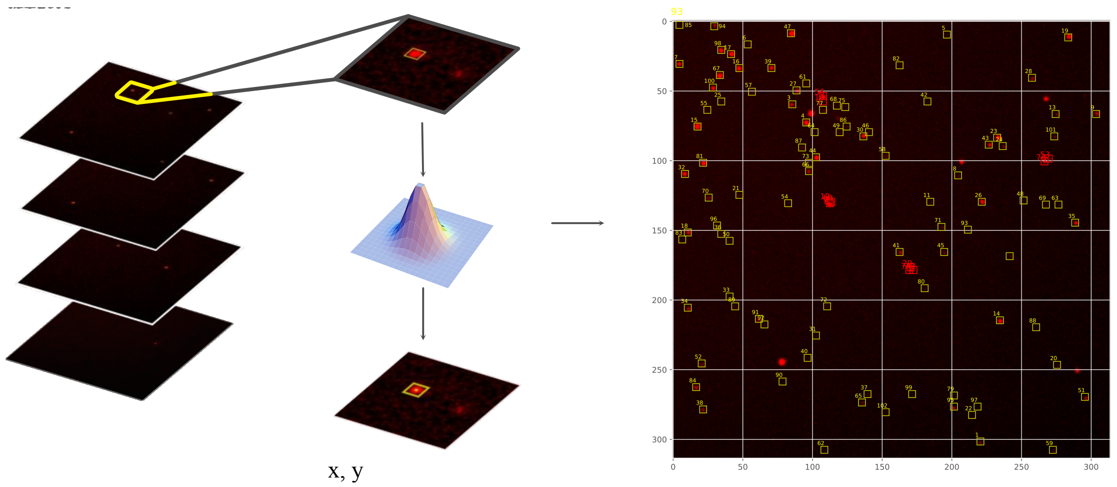

Figure S4. Localization of single molecules. Single molecules were localized using the localization module contained in the Picasso package, which uses a Gaussian fitting algorithm.[7] The obtained framewise x,y-coordinates were combined in a radius of 2 pixels and average coordinates were calculated, which were used to define a bounding box with a side length of 5 pixels. The intensity within the box was summed up to give the particle intensity for each frame.

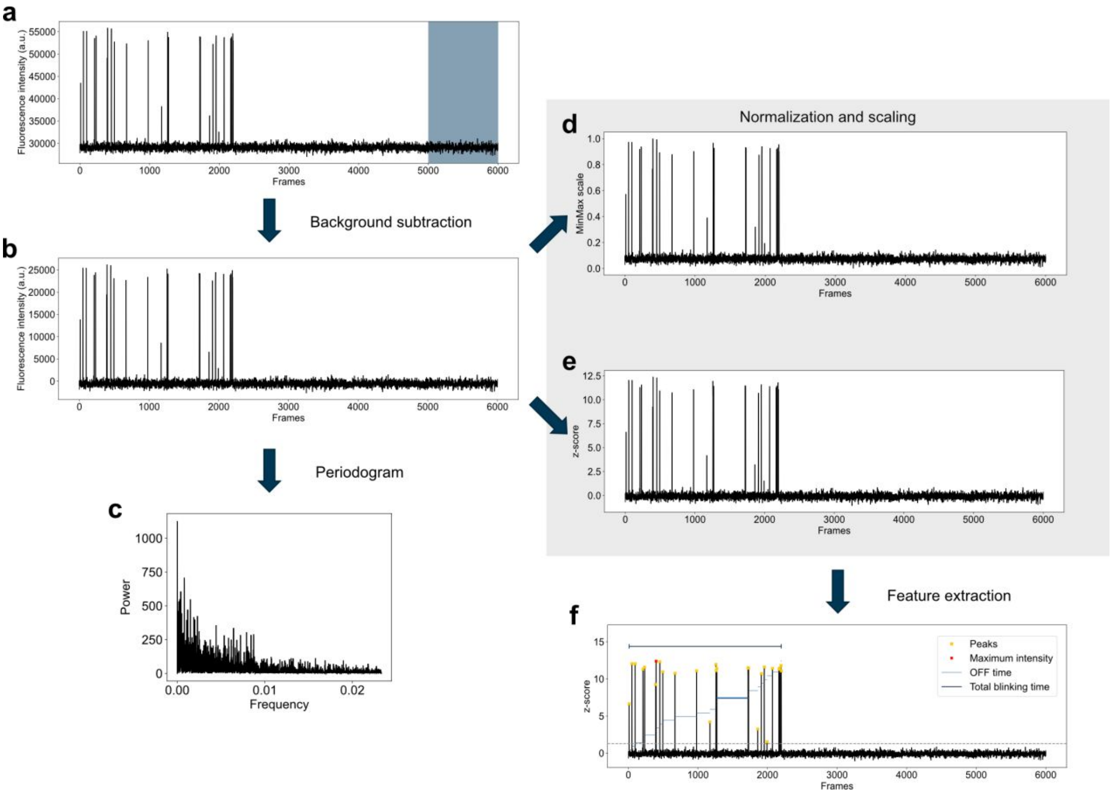

Figure S5. Signal processing after obtaining the trace from the single-molecule localization. **a**, Raw trace obtained from summing the intensity in the movie within a box of 5x5 pixels around the localization. The area of the last 500 frames is shaded in dark blue, which is used to determine the average background. **b**, The trace obtained after subtraction of the mean calculated from the last 500 frames (shaded in **a**). **c**, Example of a periodogram obtained from the background-subtracted trace, calculated using the frequency 1/(frames per second) not further used for classification. **d**, The trace obtained after min-max normalization of the background subtracted trace (b). **e**, The trace obtained after z-score normalization of the background subtracted trace (b). The traces shaded in gray were used as a direct input for the 1D-CNN and 1D-CNN-GRU models. **f**, Exemplary presentation of the trace properties, that were measured to calculate the features for classification listed in Table S3.

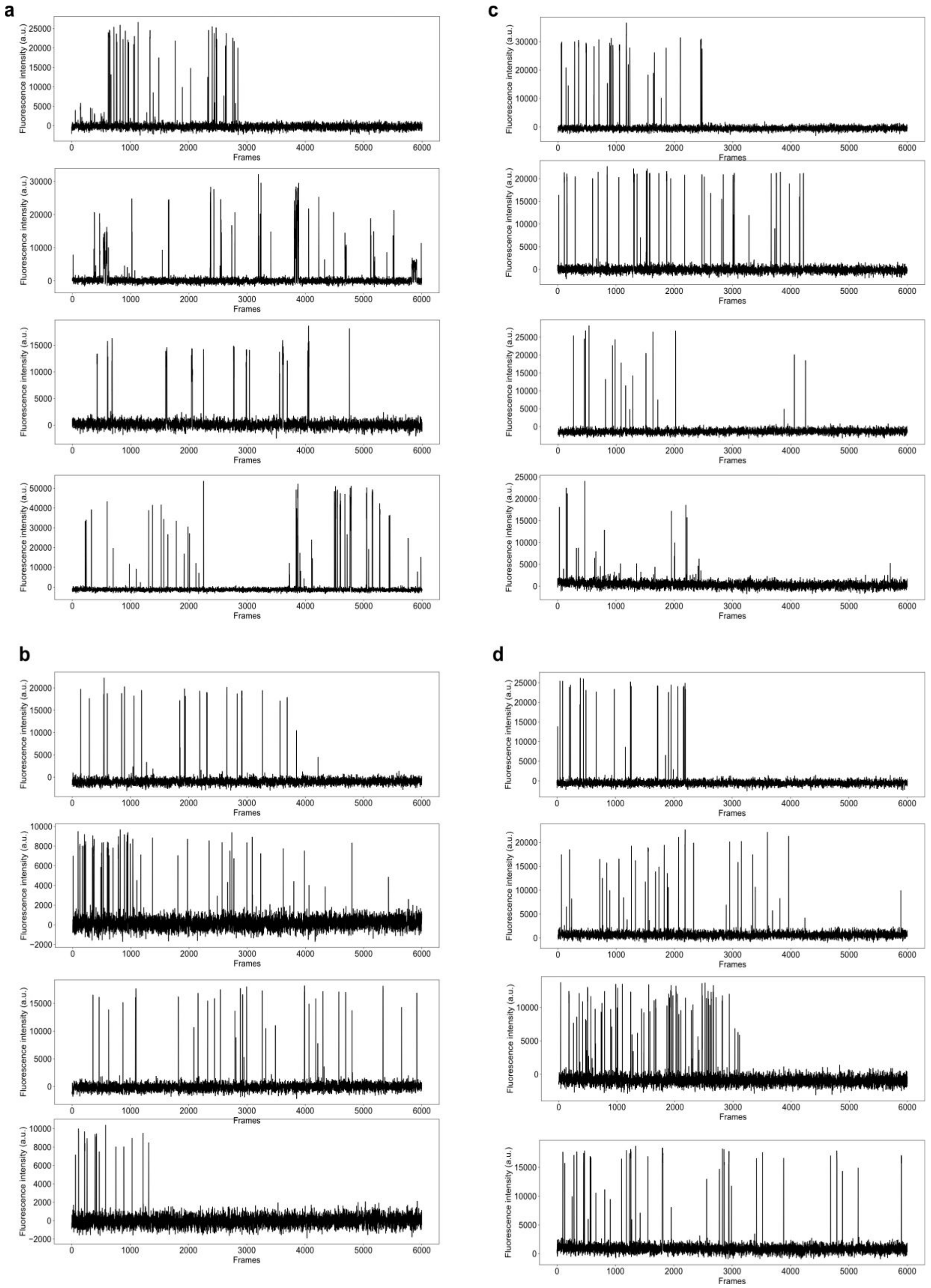

Figure S6. Randomly picked example traces of compounds after background subtraction. **a**, Traces from compound **C1**. **b**, Traces from compound **C2**. **c**, Traces from compound **C3**. **d**, Traces from compound **C4**.

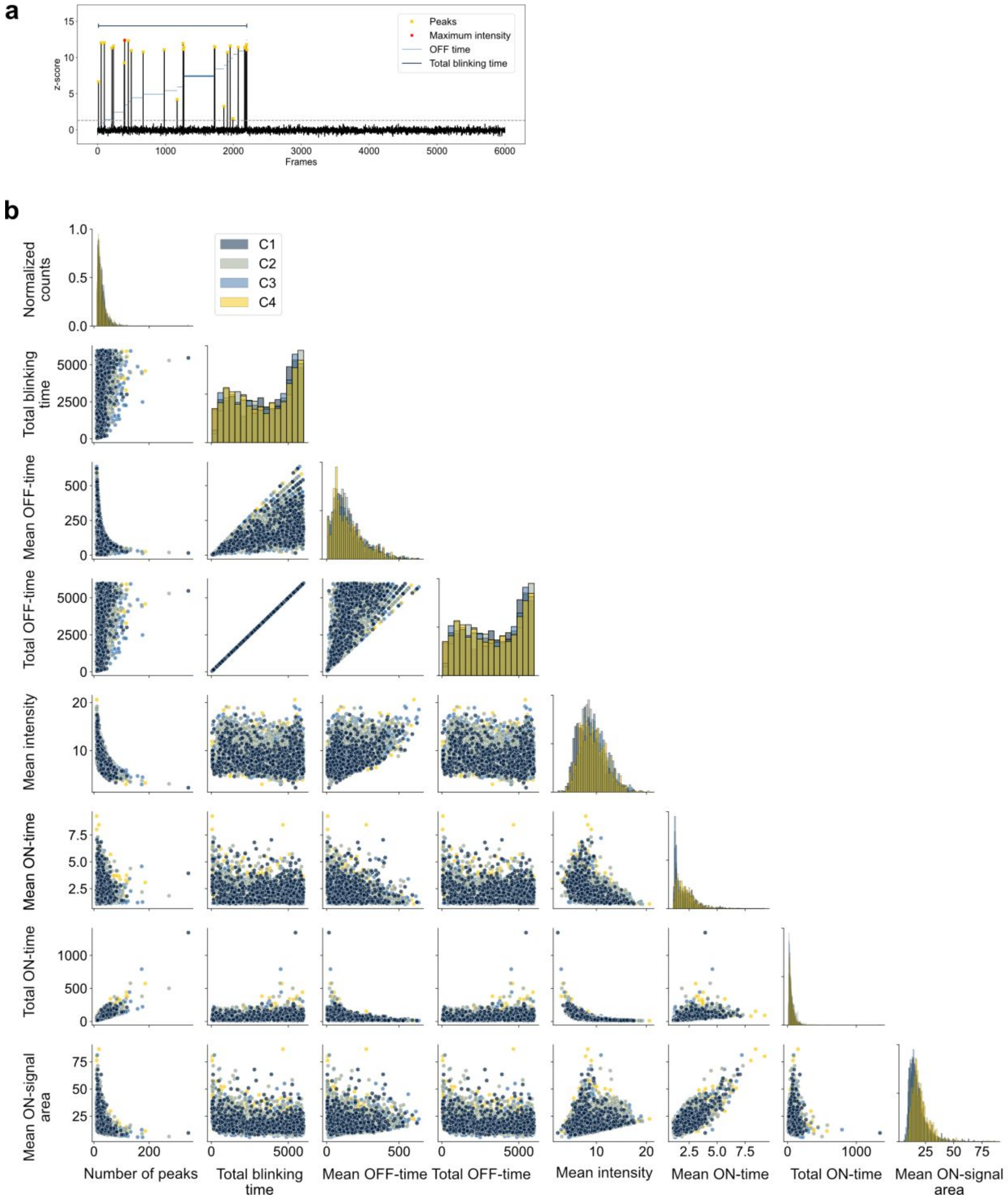

Figure S7. Initial visualization of the extracted features. **a**, Exemplary presentation of the trace properties that were measured to calculate the features for classification listed in Table S3. **b**, Examples of cross-correlation plots and histograms (diagonal), revealing no obvious clustering for the peptide classes.

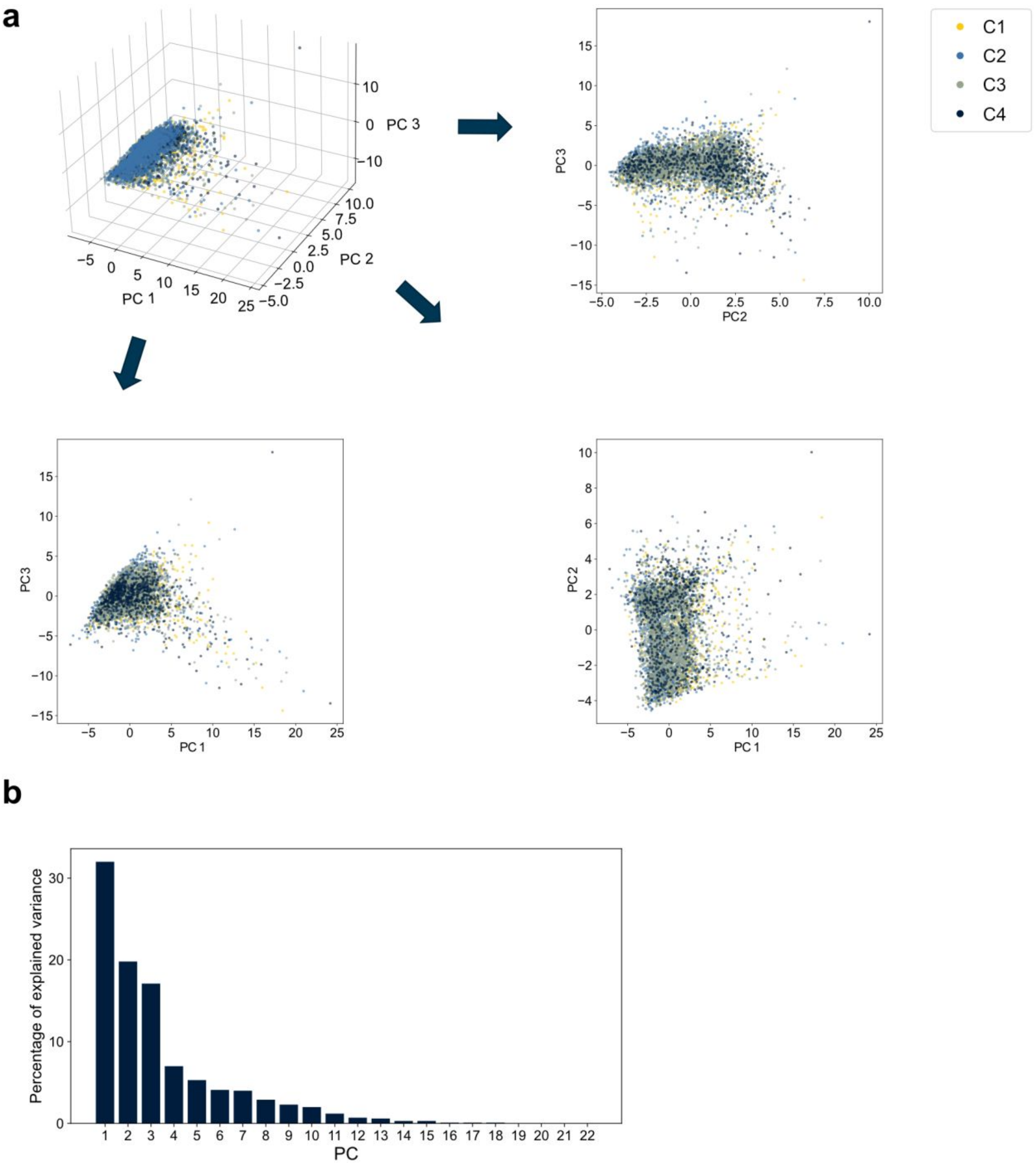

Figure S8. Outcome of the analysis using the PCA approach. **a**, Results from a PCA using 3 principle components (PCs), as a 3D view and the corresponding projections of the 3 PCs. **b**, Scree plot resulting from the PCA, shows that most variability is explained by the first 3 PCs, however, the explained variance is very low.

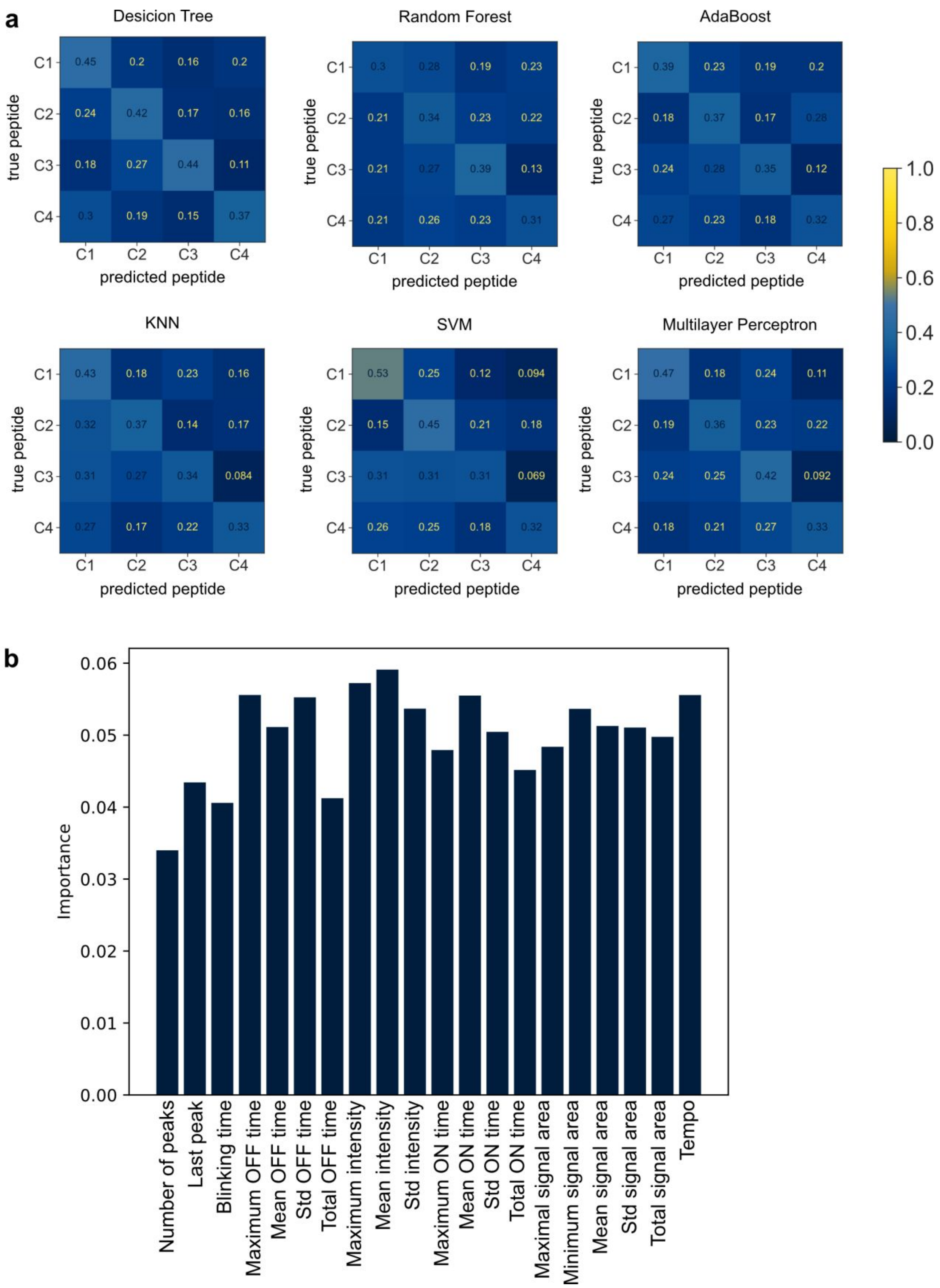

Figure S9. Classification results of a cross-validation approach with five folds using the corresponding model on the extracted feature data of compounds **C1-C4** from 479 peptide traces. **a**, Confusion matrices obtained from predictions on the test set using the corresponding model: a decision tree model, a random forest model, an AdaBoost model, a k-nearest neighbors (KNN) model, a support vector machine (SVM) model, and a multilayer perceptron. **b**, Feature importances obtain for the random forest model. We can see that most features have rather similar importance.

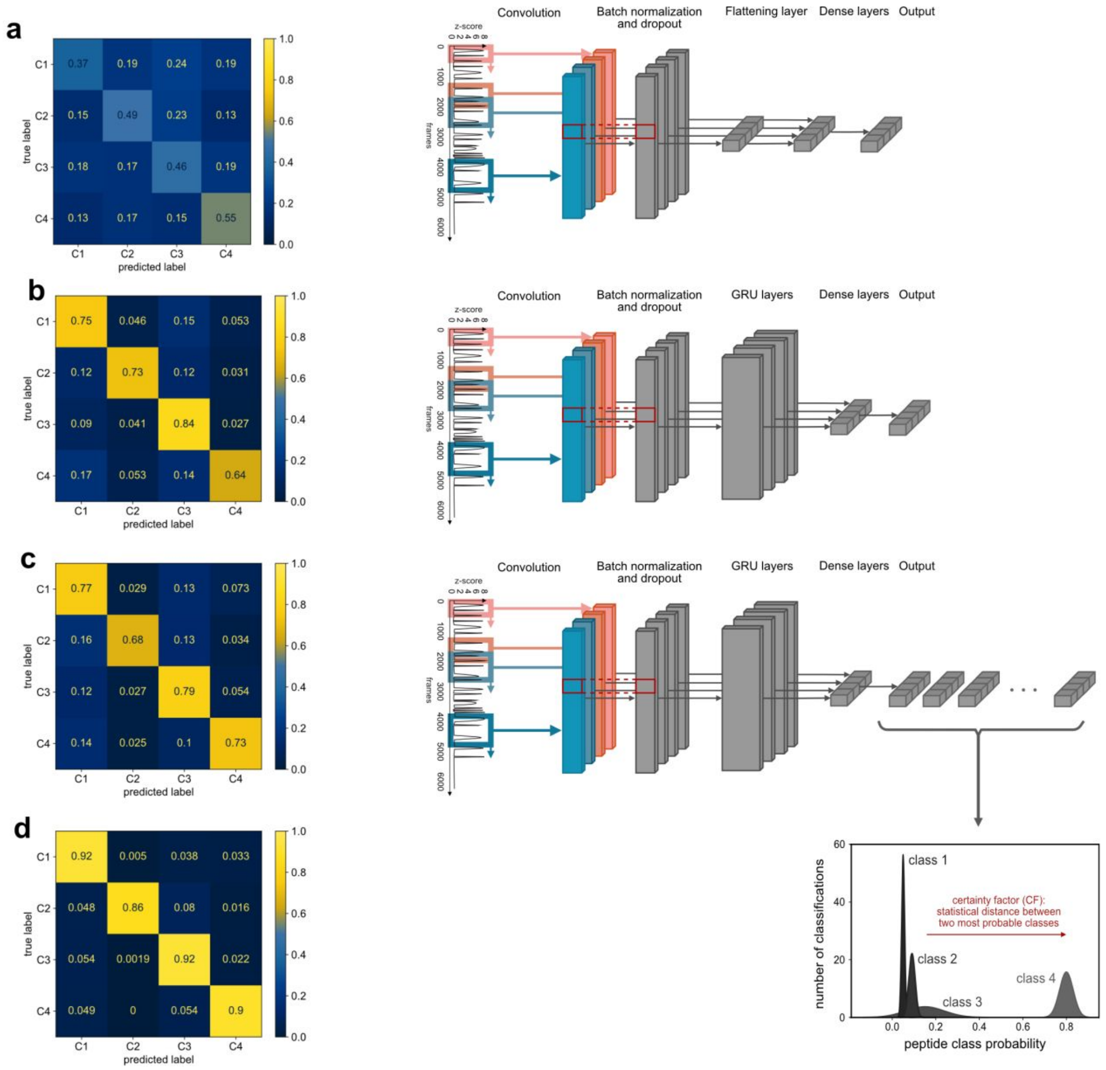

Figure S10. Classification results of the different deep-learning architectures on the same training and test sets, leading to the final results presented in this work. **a**, Confusion matrix (left) obtained using a deep learning model using only CNN layers before the dense and softmax layer (right) classifying 2380 peptide traces. **b**, Confusion matrix (left) obtained using a deep learning model using only CNN layers with GRU layers (right) classifying 2380 peptide traces. **c**, Confusion matrix (left) obtained using a deep learning model using only CNN layers with GRU layers and MC dropout upon inference (right) classifying 2380 peptide traces. **d**, Confusion matrix (left) obtained using a deep learning model using only CNN layers with GRU layers and MCD upon inference with filtering based on a CT of 0.7 (for a detailed explanation of our MCD implementation, see Figure S12). 1060 peptide traces were classified after filtering.

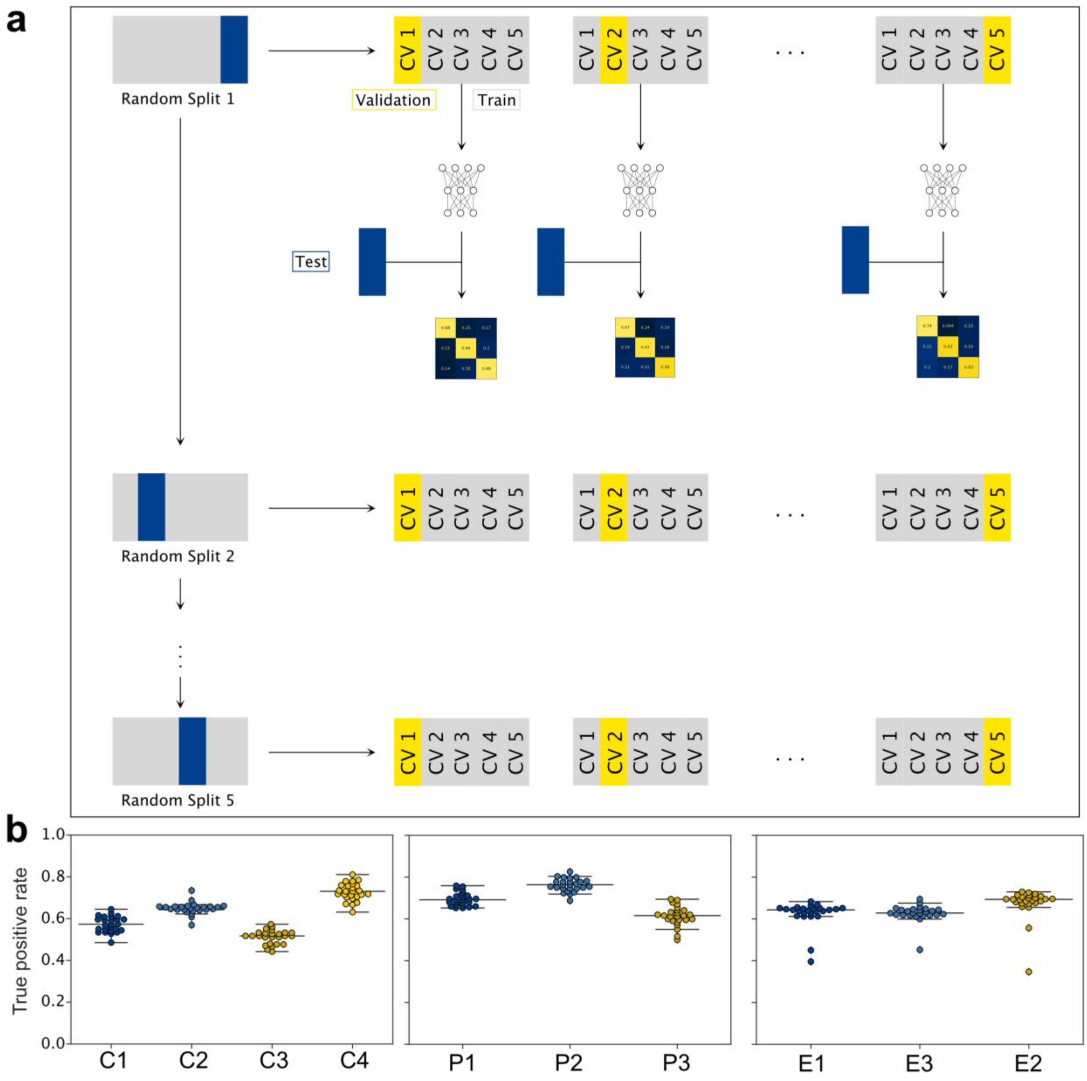

Figure S11. Nested cross-validation. **a**, Evaluation of the model (Table S7) on a dataset. The fluorescence intensity traces are split into a train/validation set and a test set. The test set is kept aside while a model is trained using the training/validation set in a five-fold cross-validation approach. Each model resulting from a cross-validation training is evaluated on the test set that split off initially. This whole process was repeated five times to reduce the influence of the initial split into a train/validation set and a test set on the evaluation. **b**, Plots of the true positive values obtained for all the 25 models trained in the five random splits with five-fold cross-validation. Values are displayed for the deterministic 1D-CNN-GRU model. The middle line represents the median of all the values, while the whiskers represent the quartiles, without outliers as determined in the Seaborn implementation.[5] For the epimers set we can observe outliers in the evaluation results, resulting from models that did not converge, most likely due to exploding gradients during training.

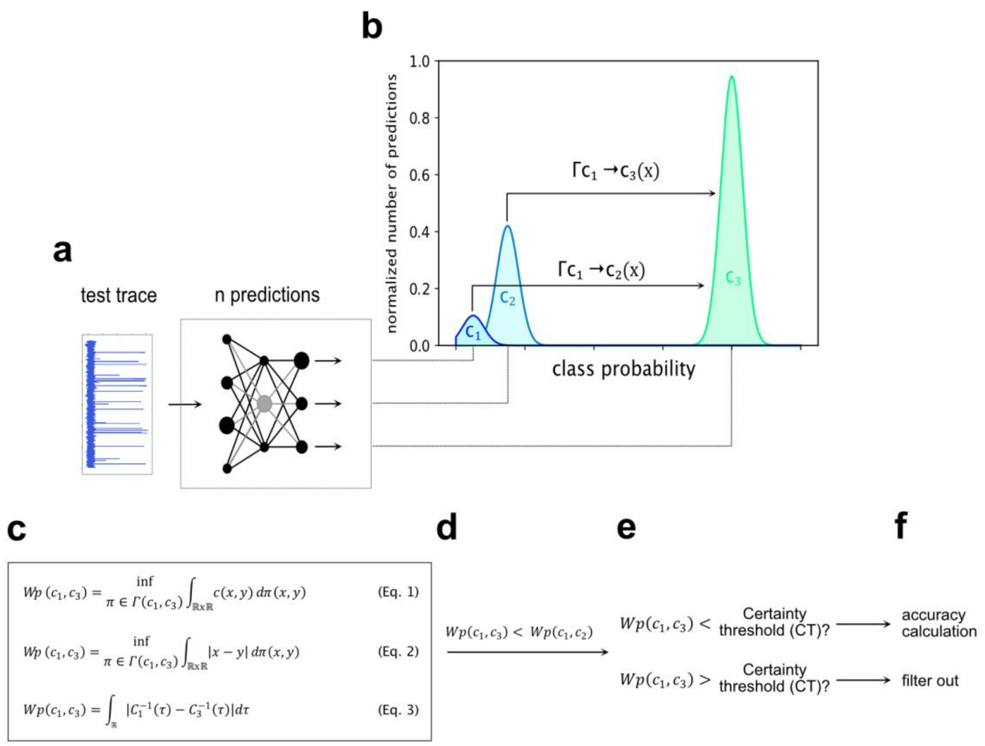

Figure S12. Principle of MCD and definition of certainty threshold (CT). **a**, To get statistics for each test trace in the test set**,** 100 predictions are calculated using the model 1D-CNN-GRU-MCD with active dropout. **b**, The Wasserstein distance between the class with the highest probability and the other classes are calculated. The Wasserstein distance, also called the earth**-**movers distance**,** represents the minimal cost of transforming one distribution into another one and therefore takes the distance and the shapes of the distributions into account. Γ represents the mapping of one distribution into the other. **c**, The intuitive understanding of the formal description of the Wasserstein distance can be described as: *π(x,y)* corresponds to the mass that is moved from *x* to *y* and *c(x,y)* the cost function, resulting in the whole cost of the transport plan π of an infinitesimally small mass to be *π(x,y)c(x,y)dxdy* and the total cost of the transport plan is the integrated form (Eq. 1). As we can see in Equation 1**,** the transport plan π is part of an ensemble of many possible plans $\Gamma(c_1, c_3)$, but the Wasserstein distance is based on the optimal transport, therefore the minimization problem must be solved. In case of the W_1 distance, which we applied for our calculations, the cost function c(x,y) corresponds to the distance that the mass is moved |x-y| (Eq. 2). The W_1 distance can be expressed in a closed form through the cumulative distribution function, which is implemented in the SciPy python library (Eq. 3).[8] **d**, To determine the relevant distance**,** all distances to the class with the highest model output are calculated and the minimal distance is saved. **e**, The minimal distance is compared to the CT, which is a number chosen by the user (0 ≤ CT ≤ 1). **f**, If the distance is larger than the CT**,** the trace is included for the accuracy calculation if not it will be discarded.

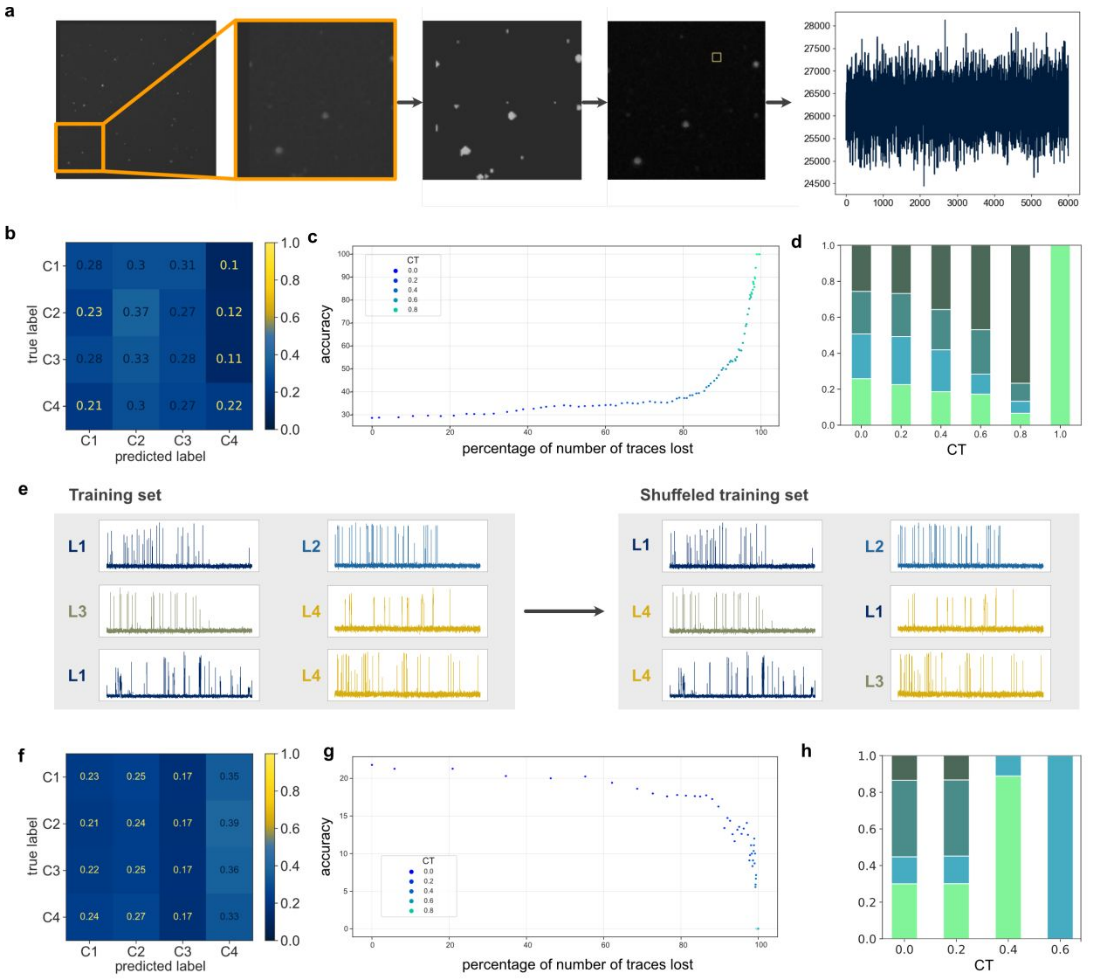

Figure S13. Control experiments to exclude learning of any eventual background information and unwanted information leaking in the model for the initial peptide set **C1**-**C4**. **a**, Extraction of background signal. A maximum projection of the movie (first and second tile) was segmented using the thresholding algorithm Yen (middle) separating signal areas from background.[12] Then an area of 5x5 pixels was chosen randomly in the background segment from which the intensity trace was extracted and given the label of the corresponding peptide in that movie. For each movie, 15 time traces were extracted. The extracted time traces were postprocessed and classified using the same steps and model architecture as the traces obtained from single-molecule localization (Figure S5). **b**, Classification obtained for the control experiment for which background noise was extracted from the movies and the corresponding label without filtering classifying 1218 traces. **c**, Accuracies obtained when filtering out a given number of traces based on their uncertainty. The color code depicts the corresponding CT (for the definition of CT, see Figure S12). **d**, The change in the composition of the test set when filtering using a stricter CT. Each color in the bar represent a different "peptide". **e**, Second control experiment in which the original labels of the training set are randomly shuffled to generate a new randomly assigned training set. **f**, Classification obtained from the control experiment with scrambled labels

without filtering using 2380 peptide traces. **e**, Accuracies obtained when filtering out a given number of traces based on their uncertainty. The color code depicts the corresponding CT. **d**, Change in the composition of the test set when filtering using a stricter CT. When the model does not learn, but rather guesses, the analysis becomes random and as soon as traces of one class are completely lost, the obtained accuracy is meaningless.

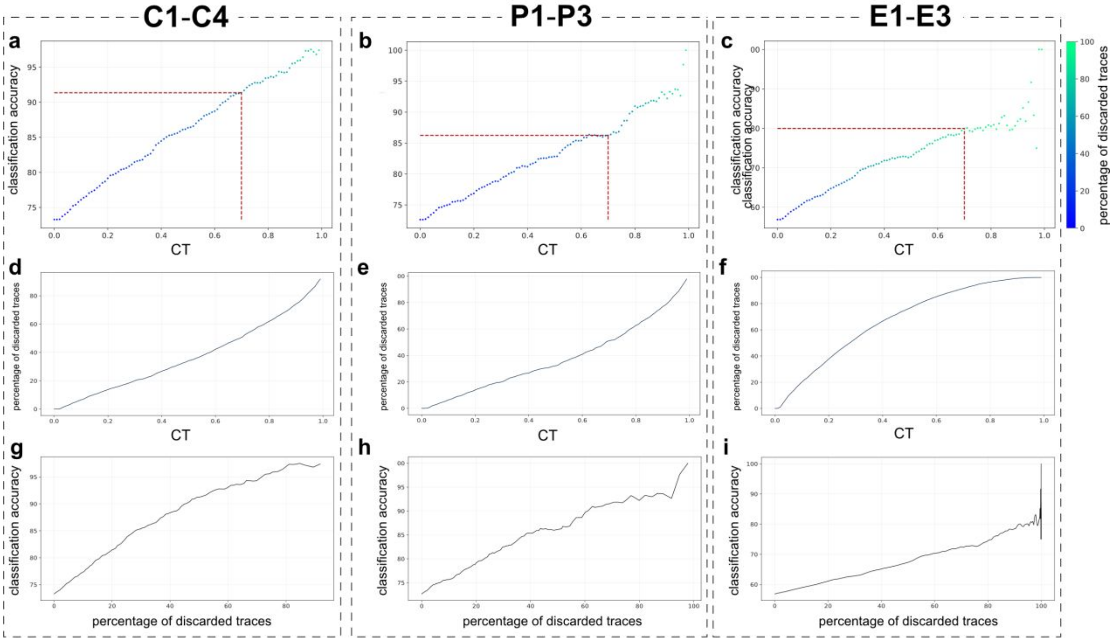

Figure S14. Certainty filtering, accuracy and discarded traces. **a-c**, Relationship between the classification accuracy and the CT used for filtering with the percentage of lost traces during filtering color-coded. **d-f**, Relationship between the classification accuracy and the CT used for filtering. **g-i**, Percentage of traces lost while filtering with the given CT.

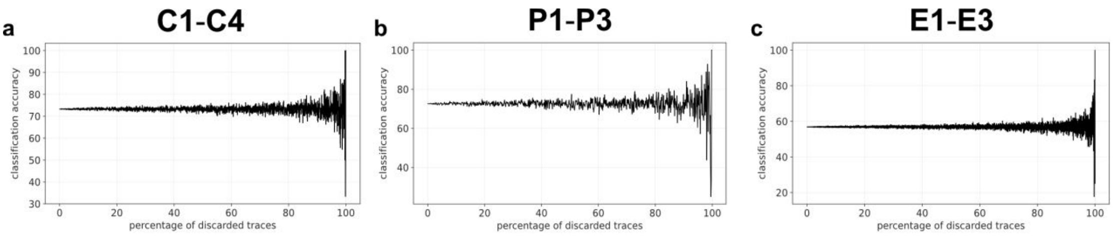

Figure S15. Classification accuracy obtained after discarding a certain percentage of random traces from the test set. This experiment shows increasingly erratic behavior upon decreasing the number of discarded traces in the test set, but no general increase in overall accuracy is observed.

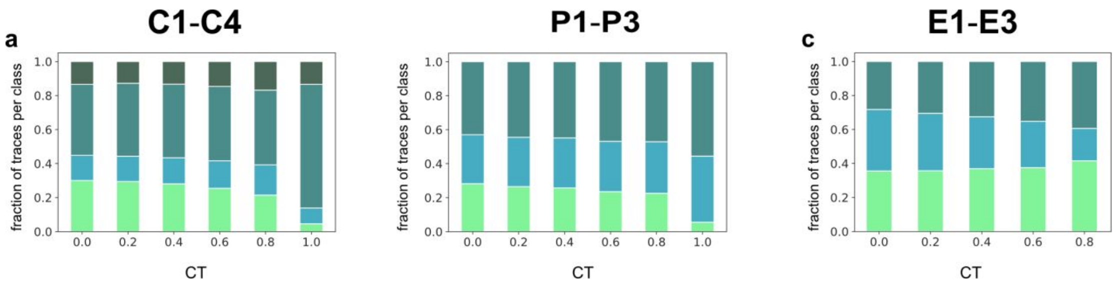

Figure S16. Composition of test set after discarding traces based on their CT value. The bars in the plots represent the percentage of traces of a class in the traces used for calculating the accuracy after filtering with a specific CT value for all peptide sets. Up to a CT value of 0.7, which was used in all cases in this work, filtering does not seem to bias the composition of the test set significantly.

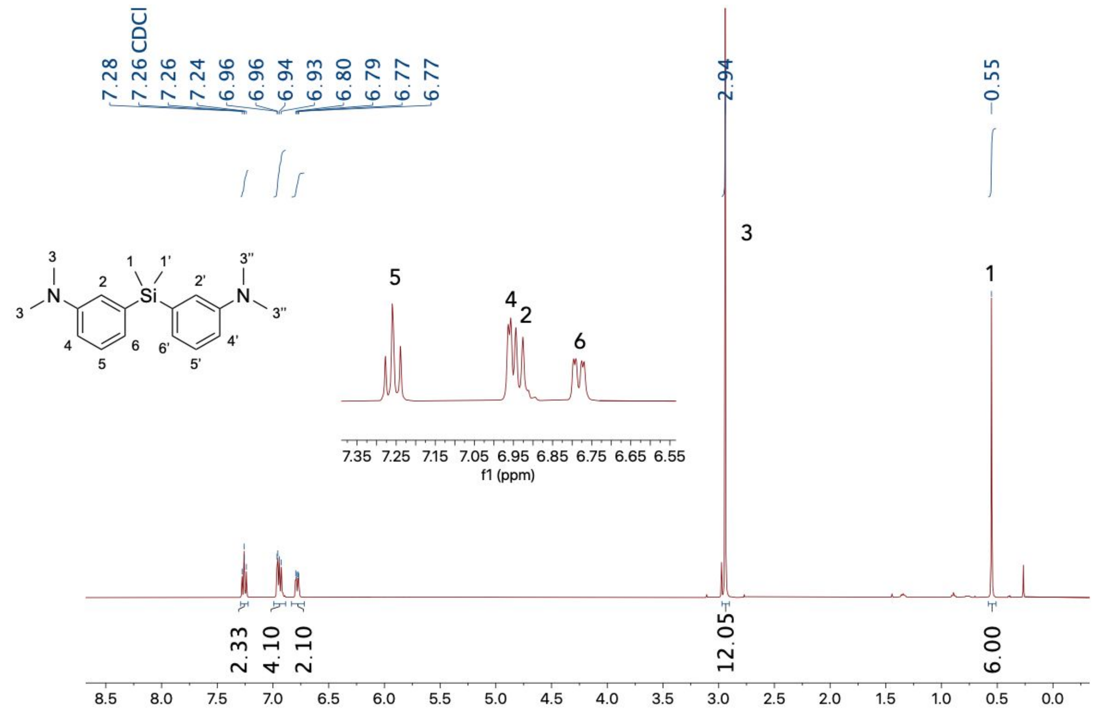

Figure S17. ^{1}H NMR ($CDCl_3$, 400 MHz) spectrum of compound **S1.**

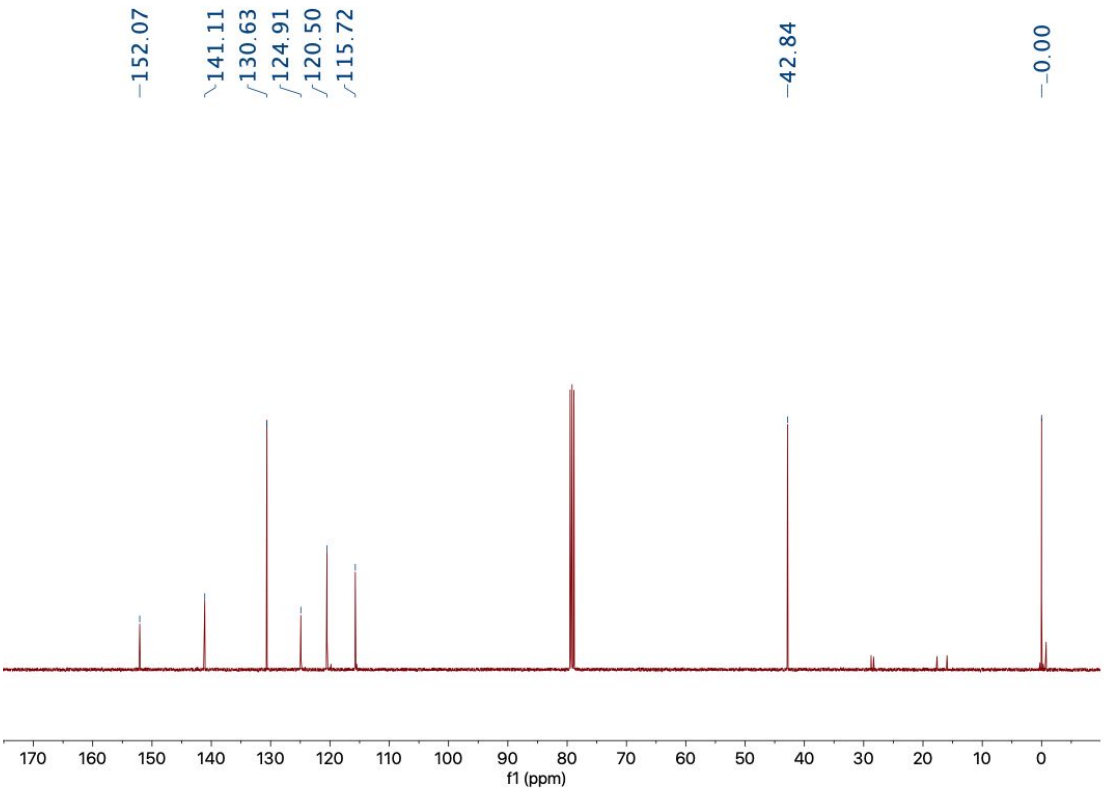

Figure S18. ^{13}C NMR ($CDCl_3$, 101 MHz) spectrum of compound **S1.**

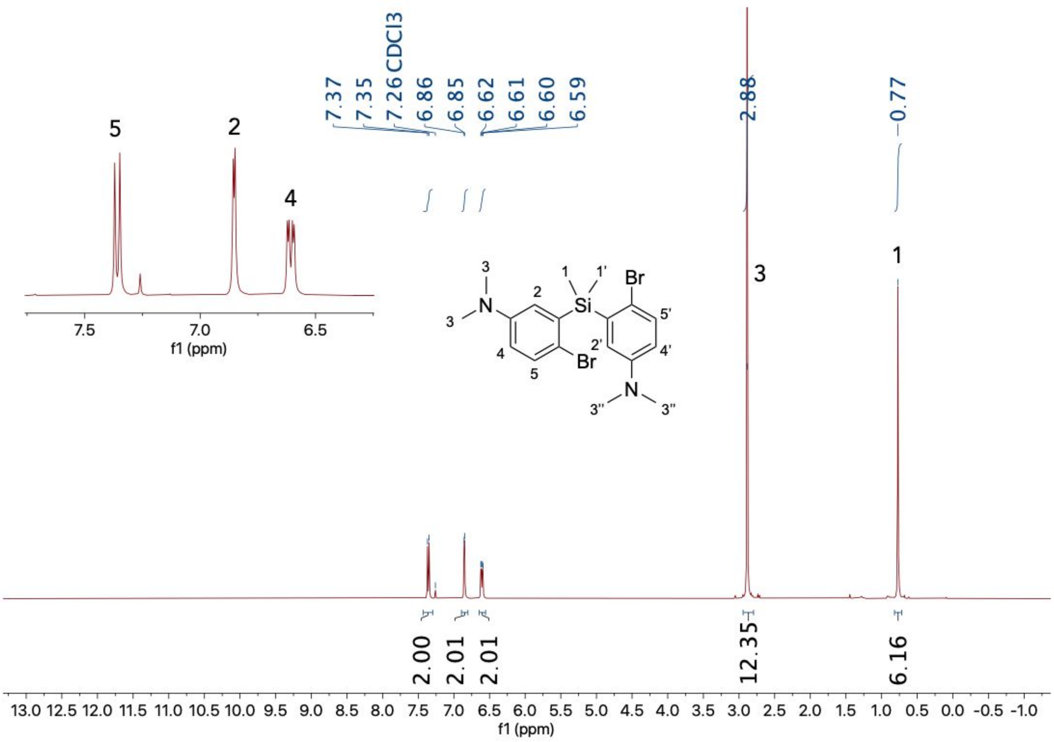

Figure S19. ^{1}H NMR ($CDCl_3$, 400 MHz) spectrum of compound **S2.**

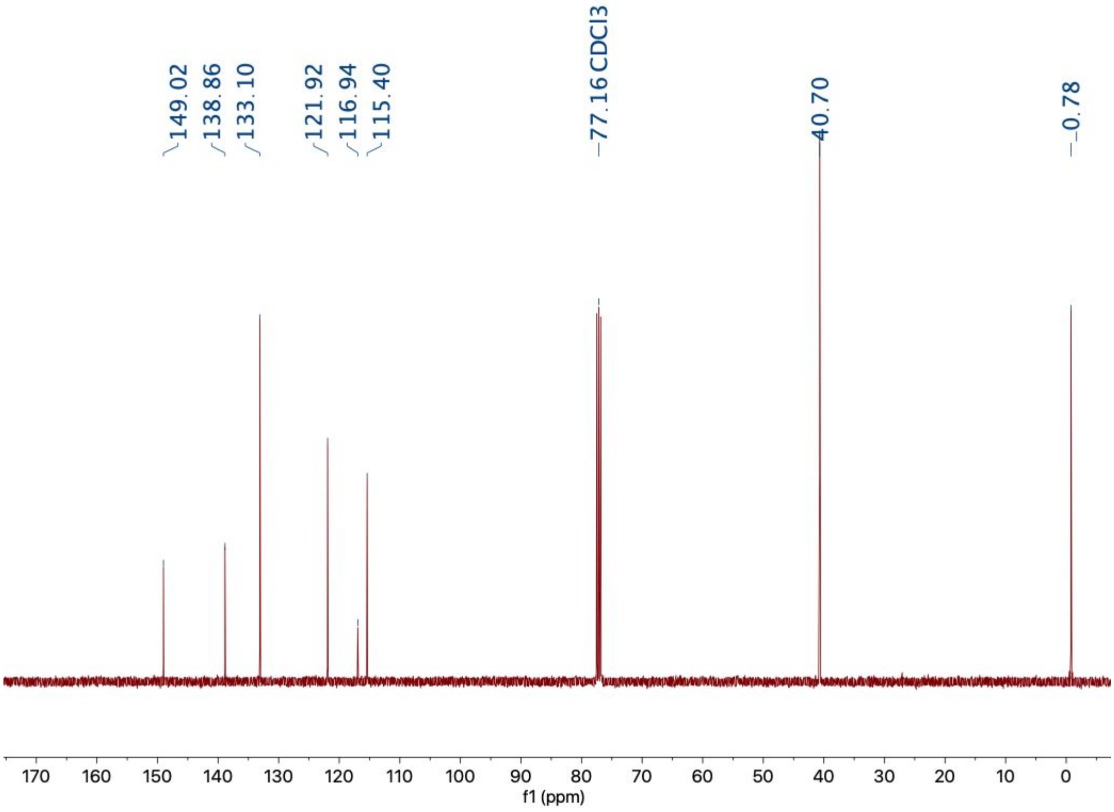

Figure S20. ^{13}C NMR ($CDCl_3$, 101 MHz) spectrum of compound **S2.**

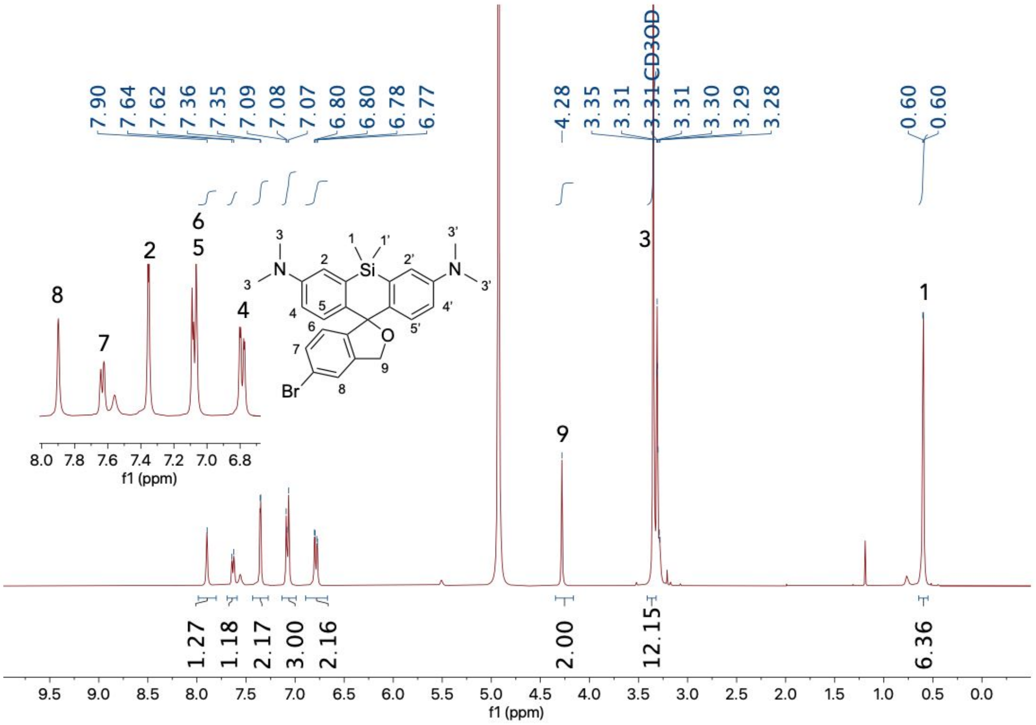

Figure S21. ^{1}H NMR ($CDCl_3$, 400 MHz) spectrum of compound **S3.**

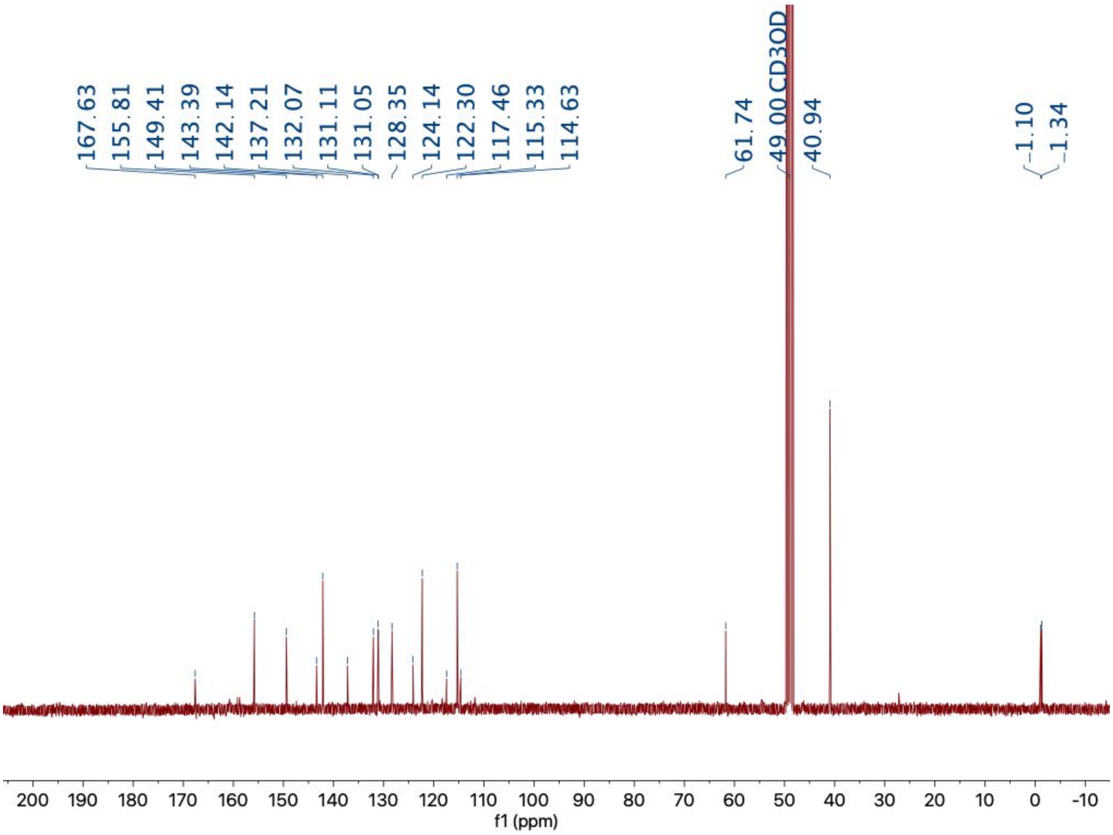

Figure S22. ^{13}C NMR ($CDCl_3$, 101 MHz) spectrum of compound **S3.**

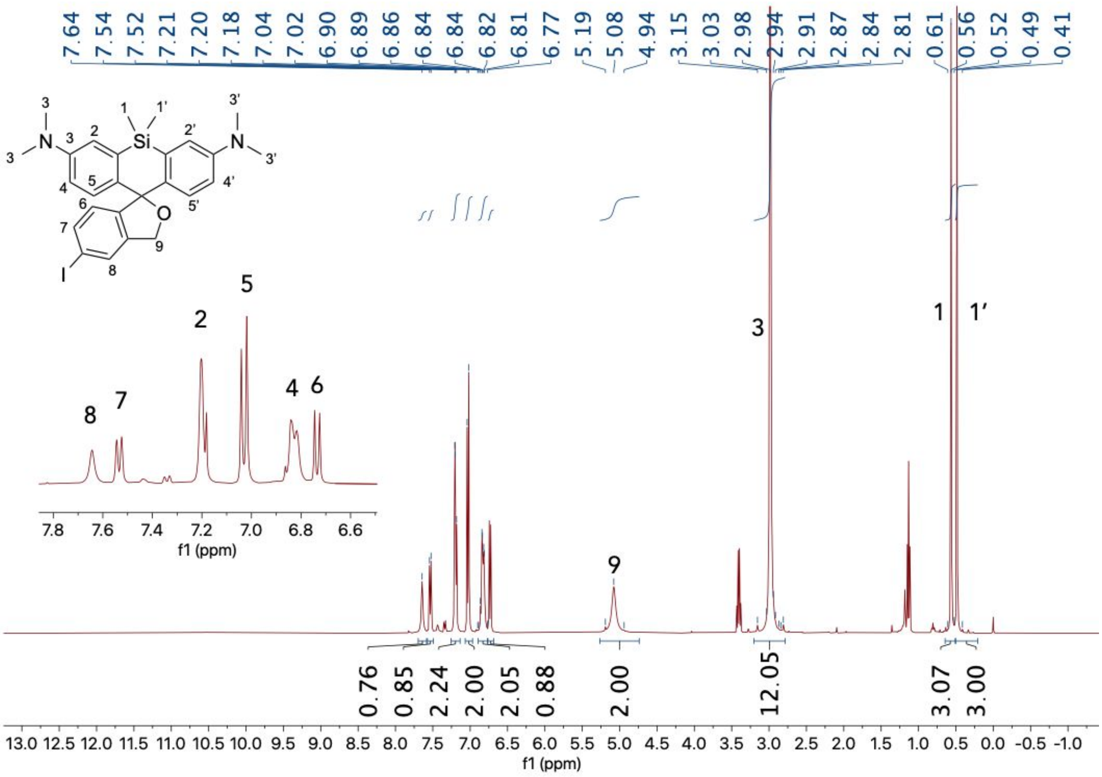

Figure S23. ^{1}H NMR ($CDCl_3$, 400 MHz) spectrum of compound **S4.**

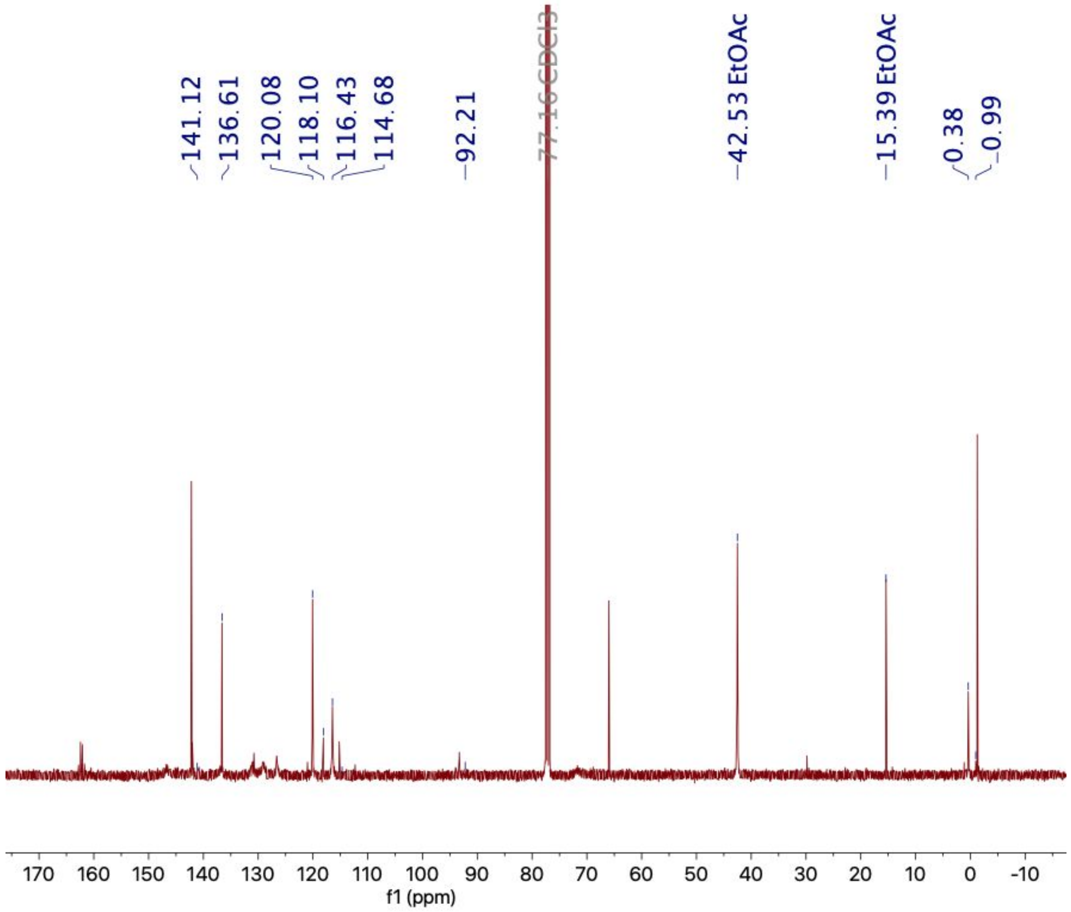

Figure S24. ^{13}C NMR ($CDCl_3$, 101 MHz) spectrum of compound **S4.**

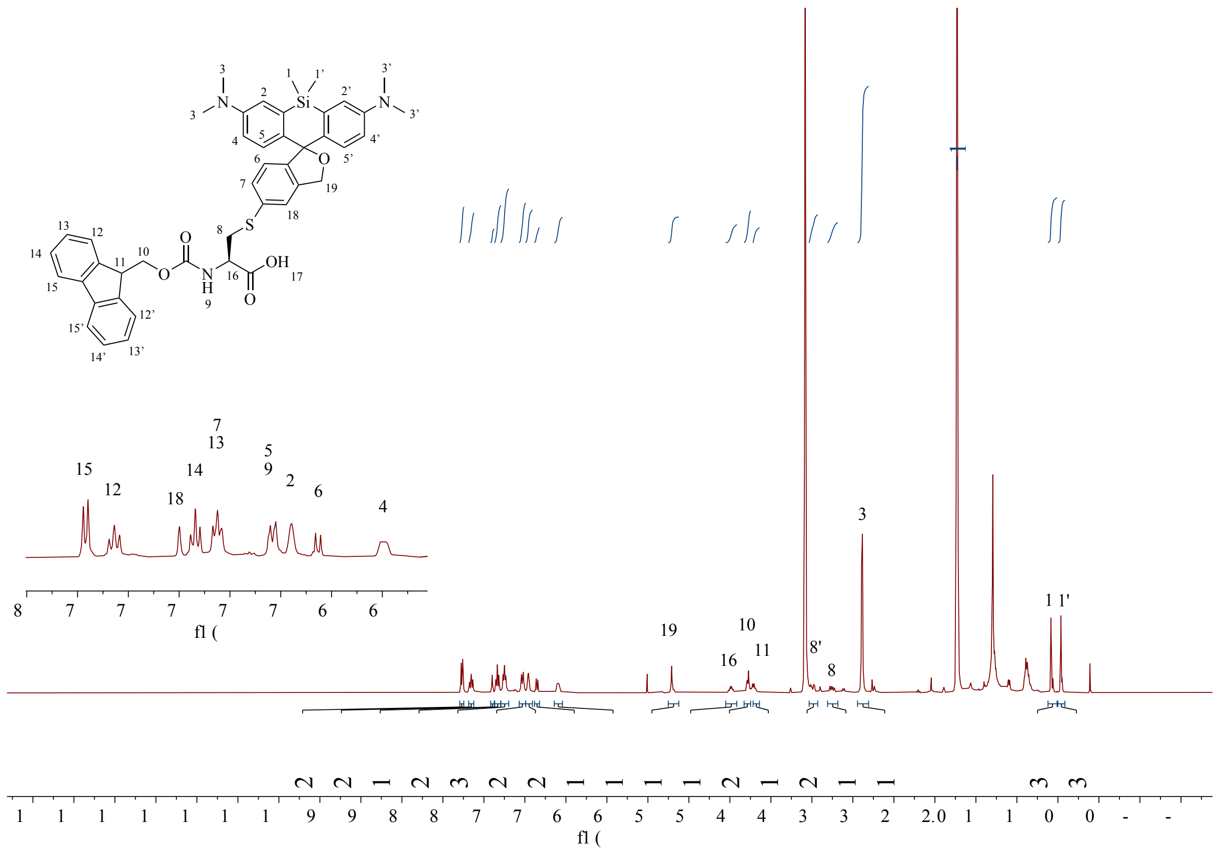

Figure S25. ^{1}H NMR (THF, 400 MHz) spectrum of compound **S5.**

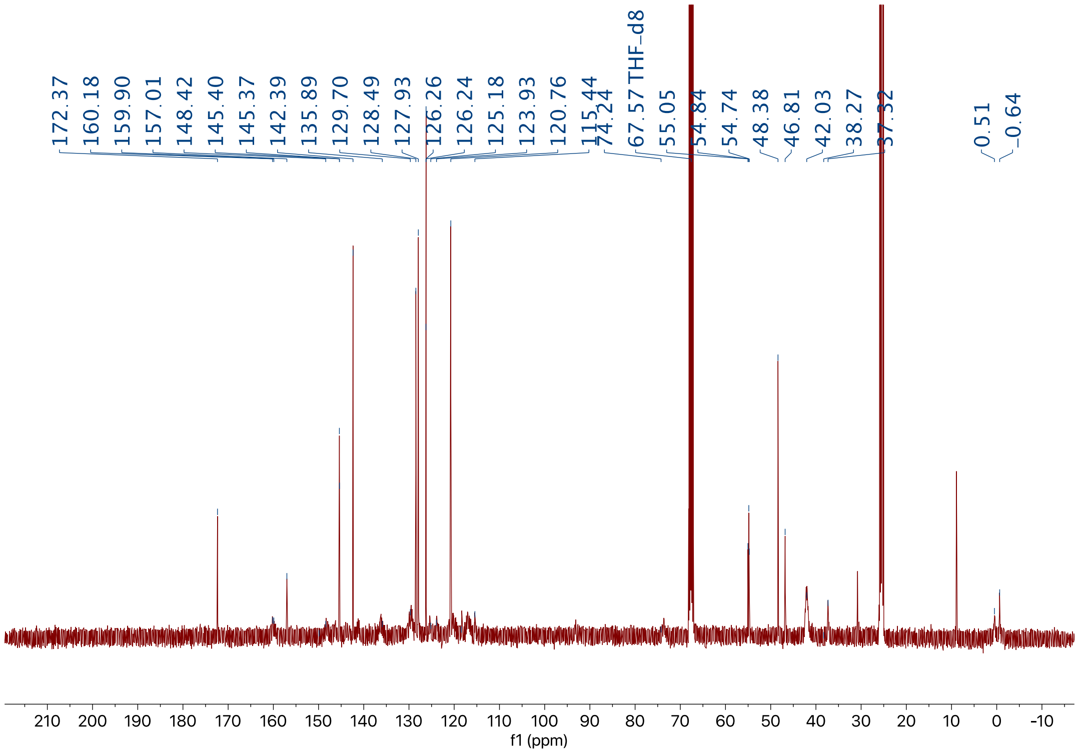

Figure S26. ^{13}C NMR (THF, 101 MHz) spectrum of compound **S5**.

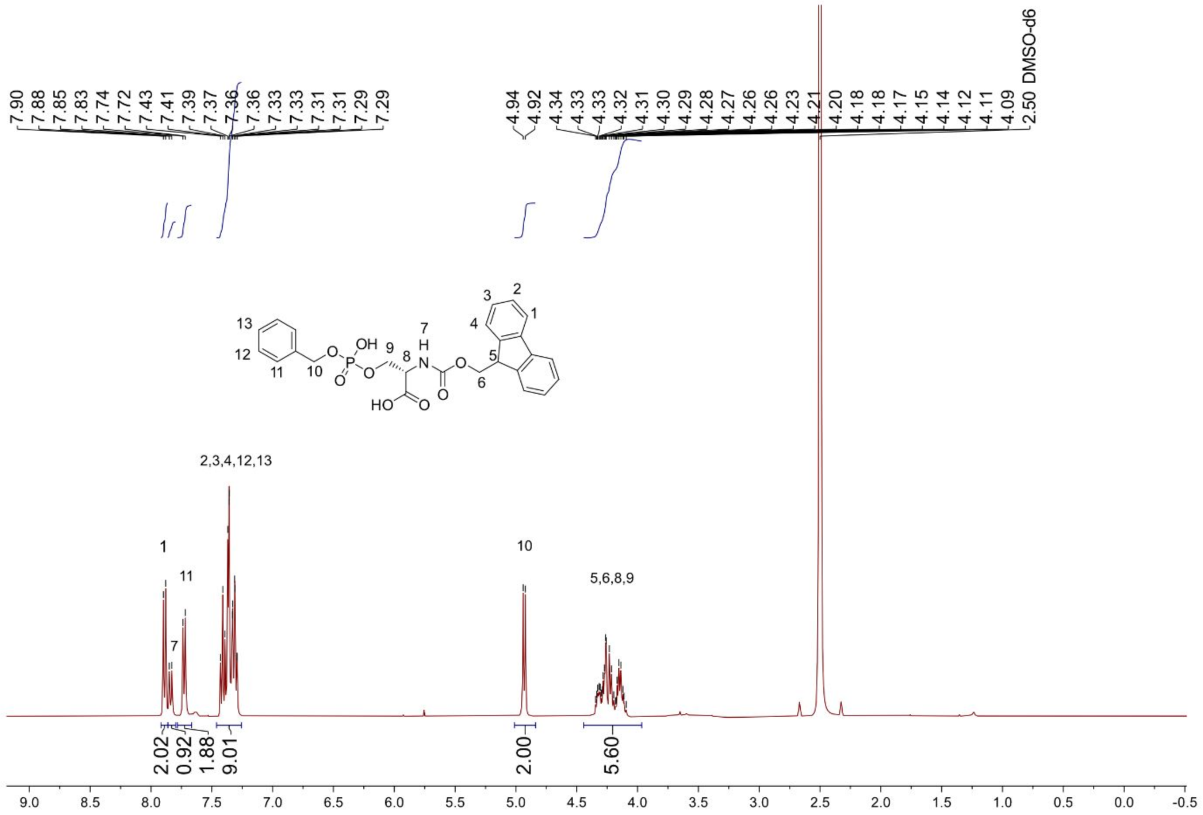

Figure S27. ^{1}H NMR (DMSO-d_6, 400 MHz) spectrum of compound **S6**.

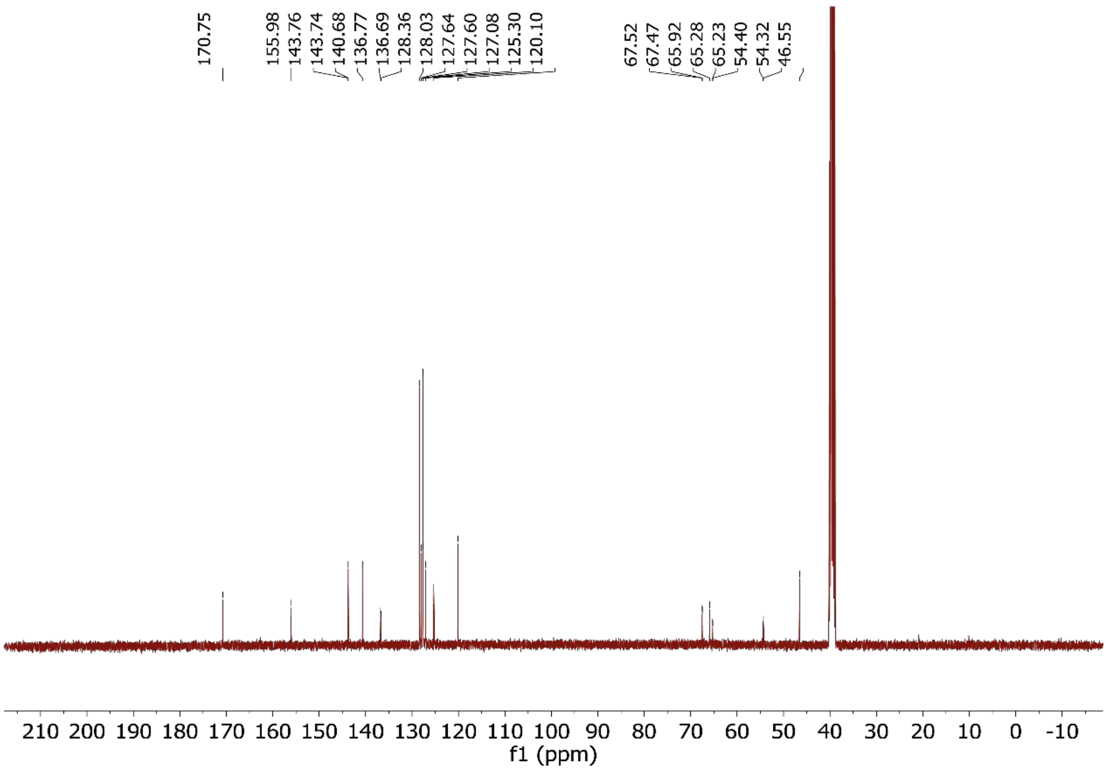

Figure S28. ^{13}C NMR (DMSO-d_6, 101 MHz) spectrum of compound **S6**.

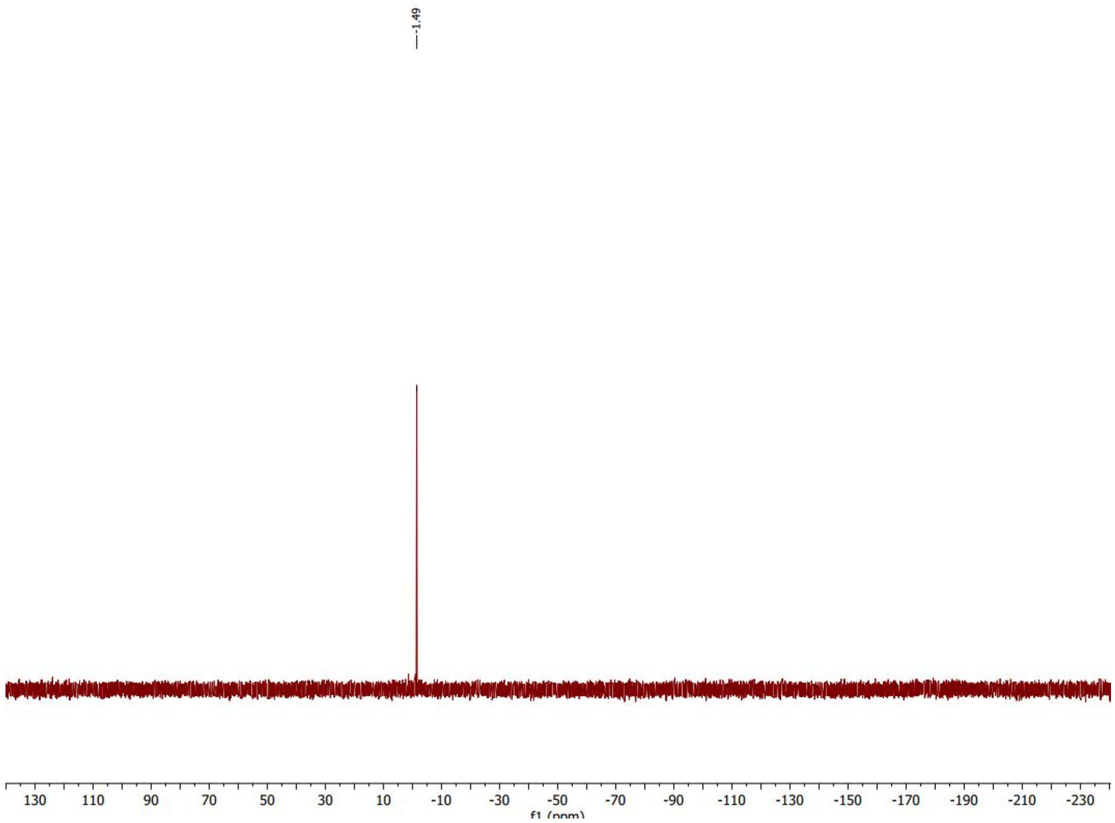

Figure S29. ^{31}P NMR (DMSO-d_6, 162 MHz) spectrum of compound **S6**.

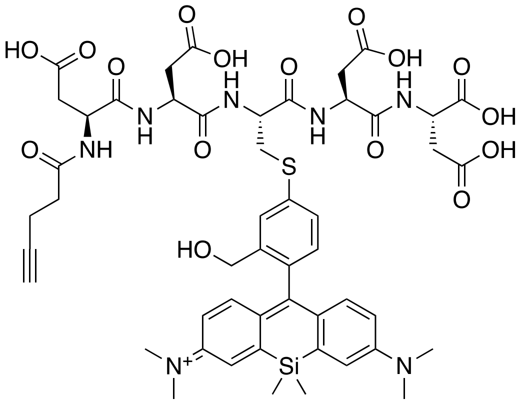

Pentynyl-DDC(HMSiR)DD-OH

HRMS (ESI): *m/z* calcd. $[C_{50}H_{61}N_7O_{16}SSi]^{+2}$: 537.6827; found 537.6832.

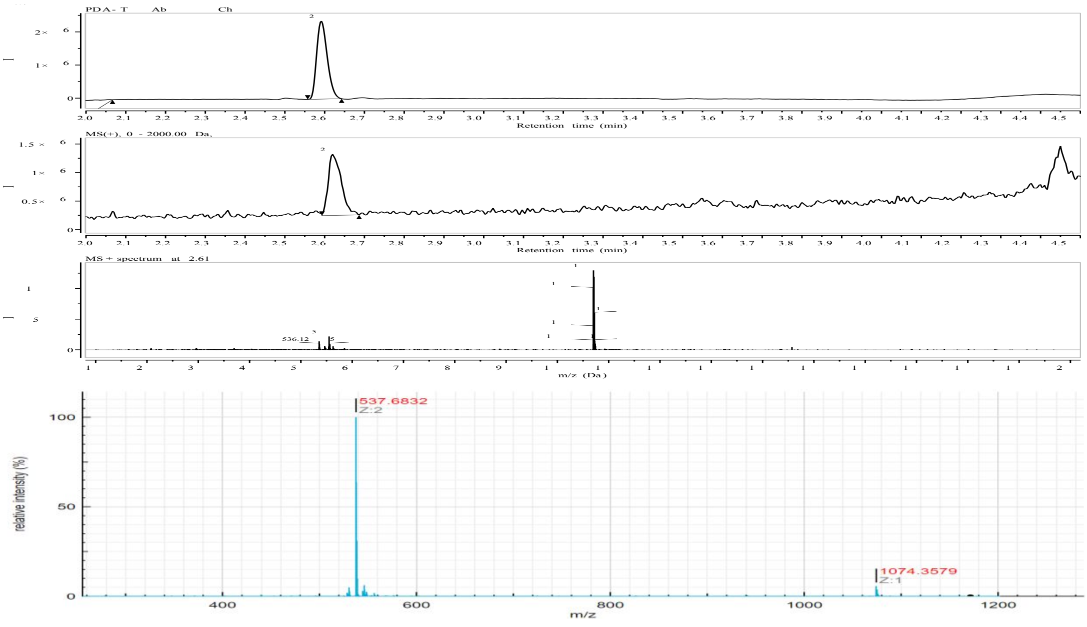

Figure S30. Structure, condensed formula, exact mass and LC-MS traces and extracted peak of peptide **C1.**

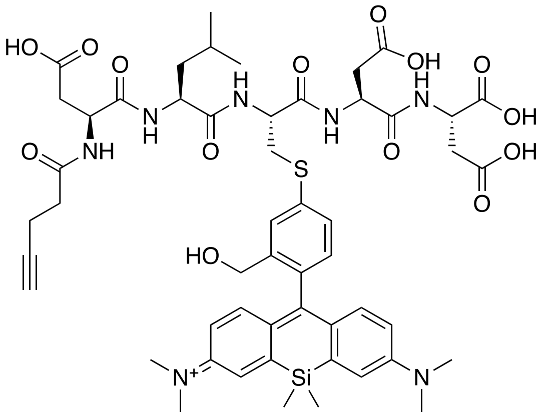

Pentynyl-DLC(HMSiR)DD-OH

HRMS (ESI/QTOF): *m/z* calcd. $[C_{52}H_{67}N_7O_{14}SSi]^{+2}$: 536.7113; found 536.7108.

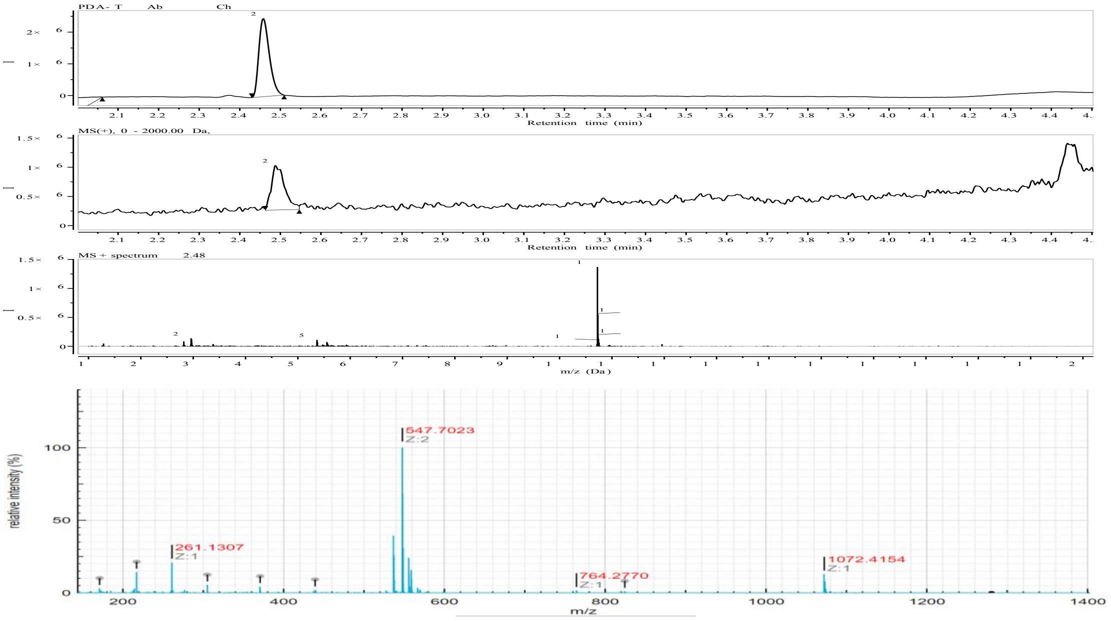

Figure S31. Structure, condensed formula, exact mass and LC-MS traces and extracted peak of peptide **C2.**

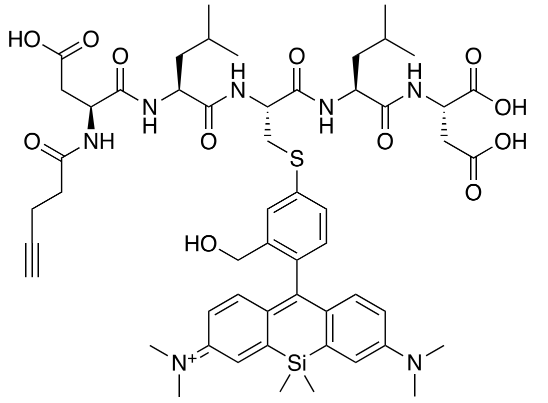

Pentynyl-DLC(HMSiR)LD-OH

HRMS (ESI): *m/z* calcd. $[C_{54}H_{73}N_7NaO_{12}SSi]^{+2}$: 546.7308; found 546.7318.

.

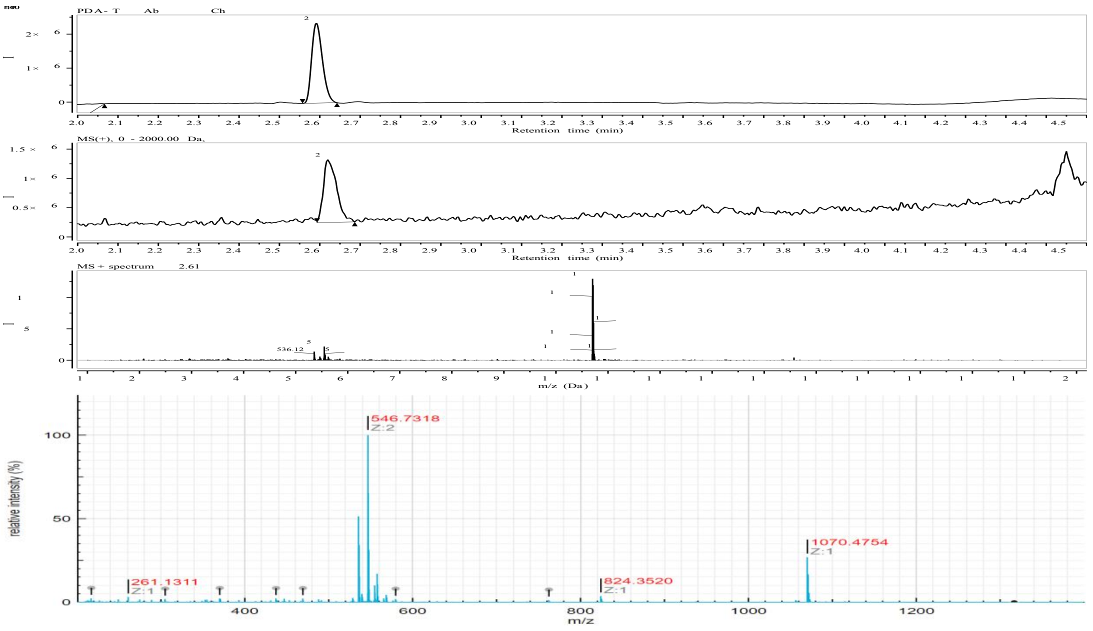

Figure S32. Structure, condensed formula, exact mass and LC-MS traces and extracted peak of peptide **C3**.

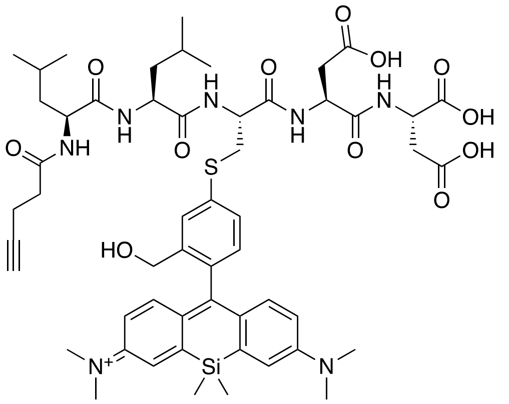

Pentynyl-LLC(HMSiR)DD-OH

HRMS (ESI/QTOF): *m/z* calcd. $[C_{54}H_{73}N_7O_{12}SSi]^{+2}$: 535.7398, found: 535.7408.

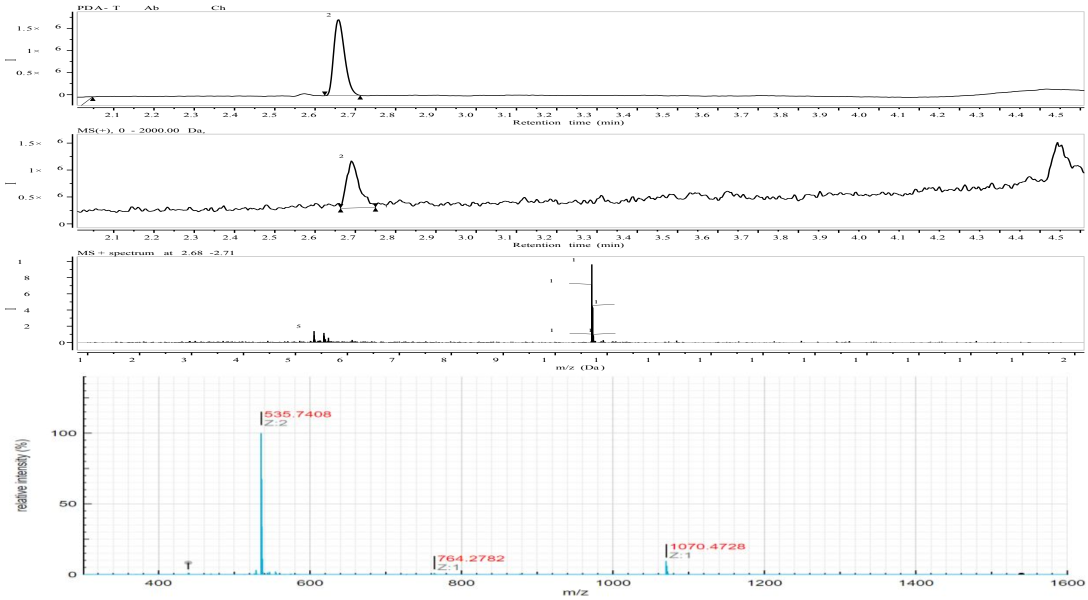

Figure S33. Structure, condensed formula, exact mass and LC-MS traces and extracted peak of peptide **C4.**

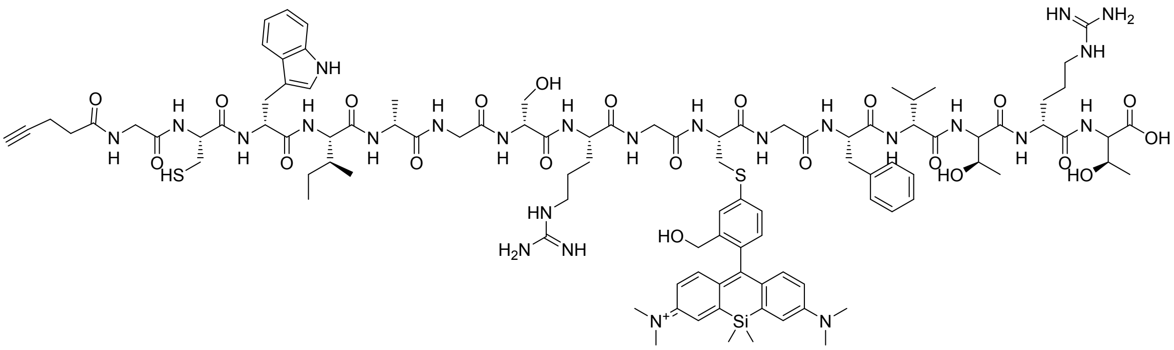

Pentynyl-GCWIAGSRGC(HMSiR)GFVTRT-OH

HRMS (ESI): *m/z* calcd. $[C_{102}H_{147}N_{25}O_{22}S_2Si]^{+3}$: 722.0116; found: 722.0130.

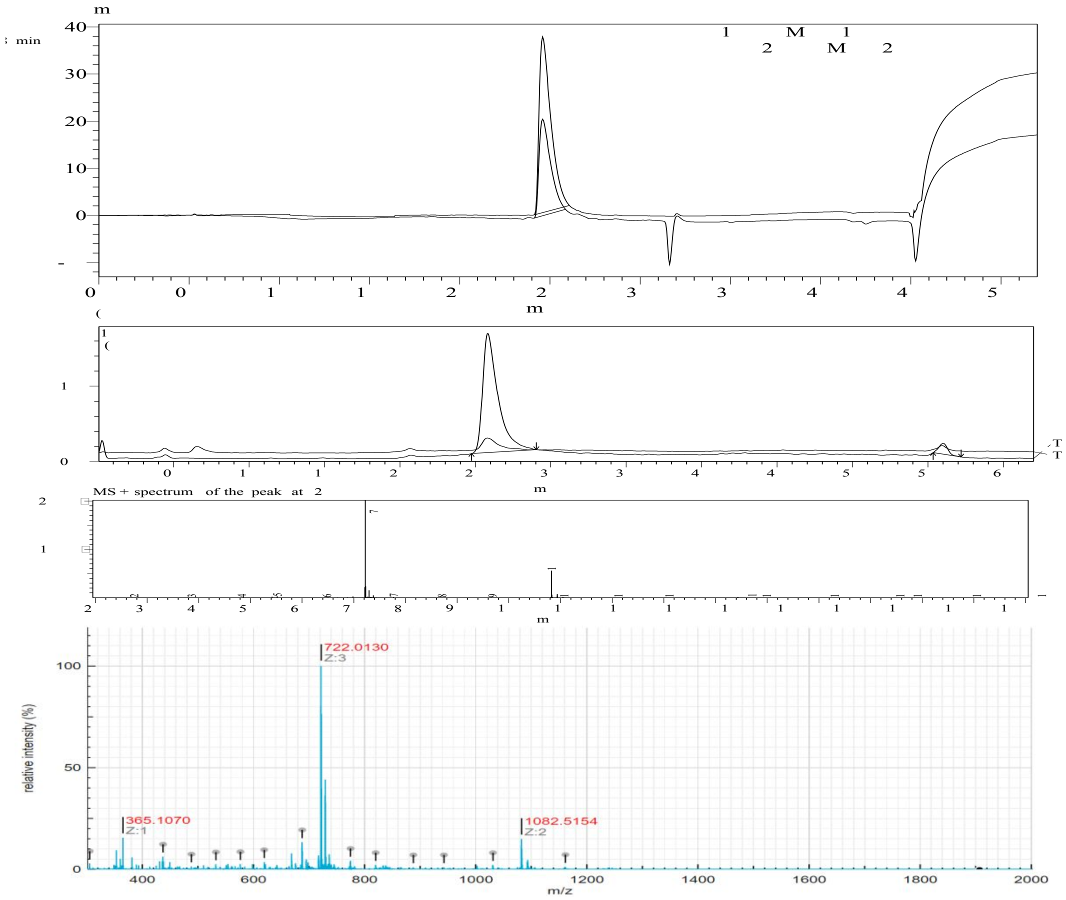

Figure S34. Structure, condensed formula, exact mass and LC-MS traces and extracted peak of peptide **E1.**

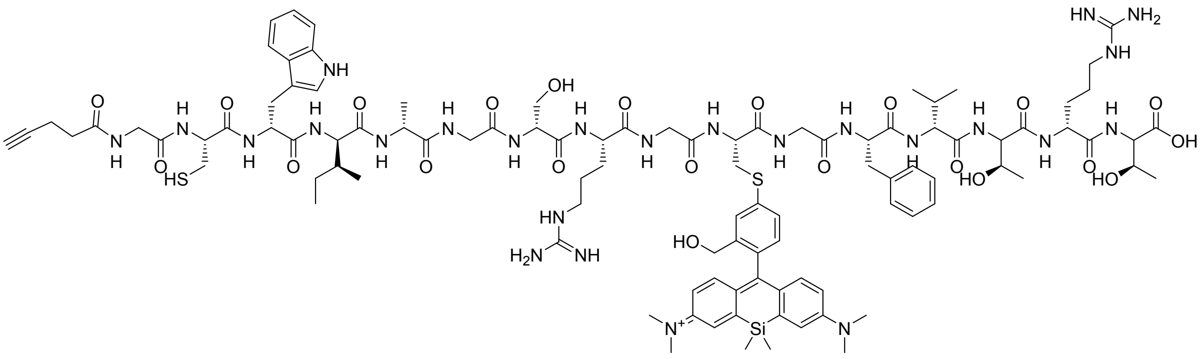

Pentynyl-GCWiAGSRGC(HMSiR)GFVTRT-OH

HRMS (ESI): m/z calcd. $[C_{102}H_{147}N_{25}O_{22}S_2Si]^{+3}$: 722.0116; found: 722.0104.

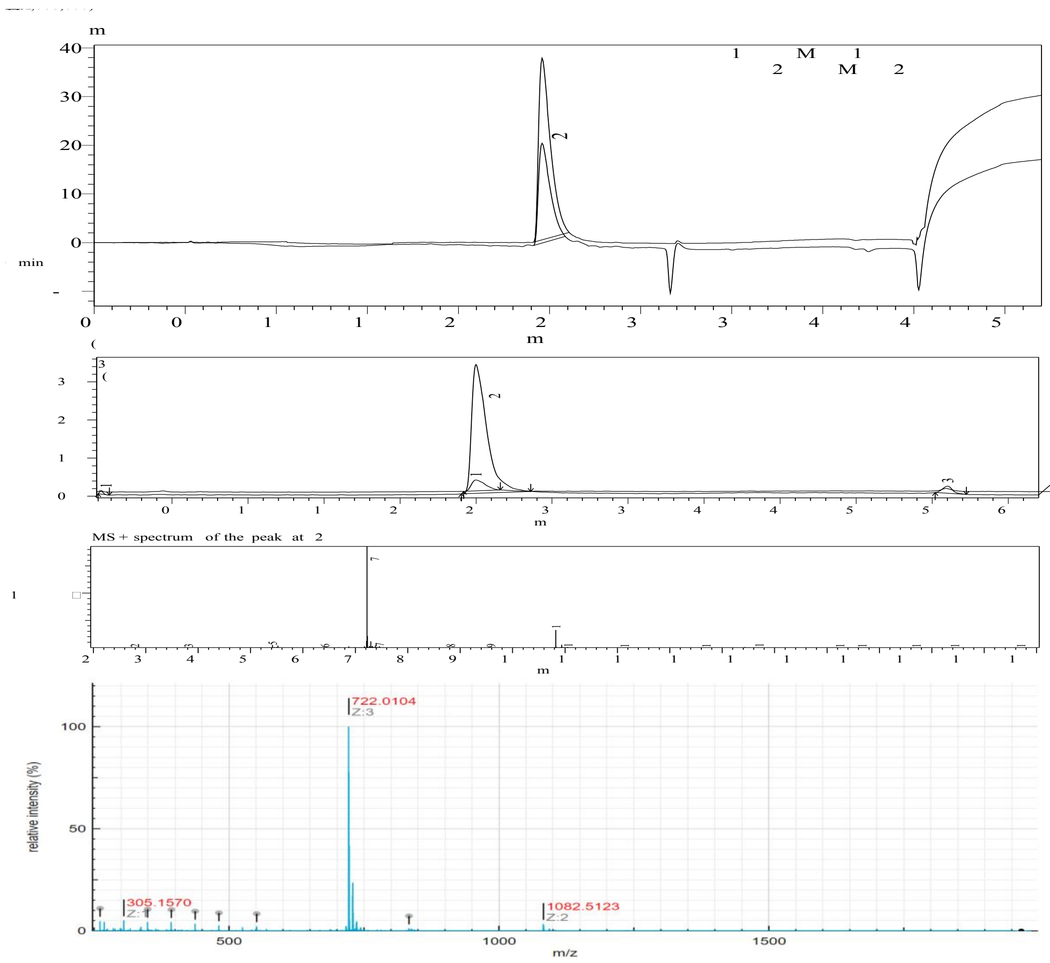

Figure S35. Structure, condensed formula, exact mass and LC-MS traces and extracted peak of peptide **E2**.

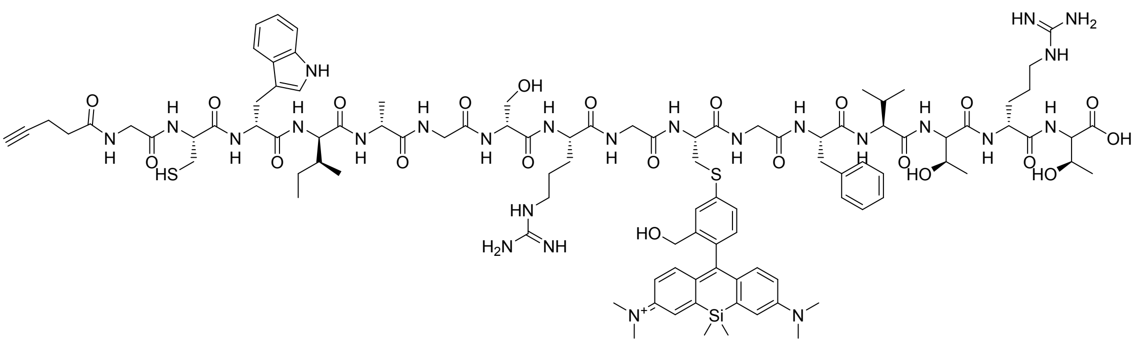

Pentynyl-GCWiAGSRGC(HMSiR)GFvTRT-OH
HRMS (ESI): *m/z* calcd. $[C_{102}H_{147}N_{25}O_{22}S_2Si]^{+3}$: 722.0116; found: 722.0128.

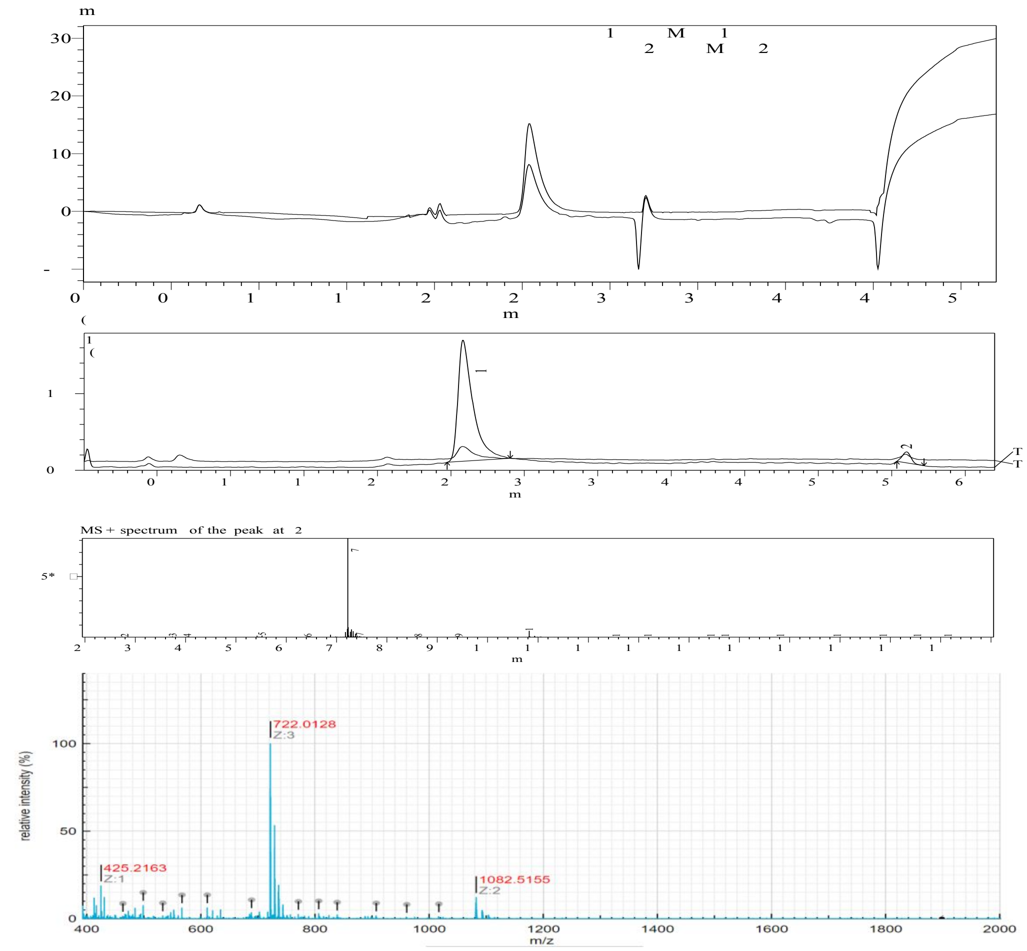

Figure S36. Structure, condensed formula, exact mass and LC-MS traces and extracted peak of peptide **E3**.

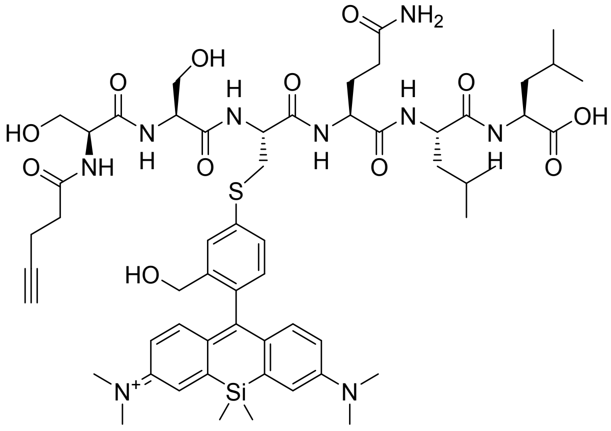

Pentynyl-SSC(HMSiR)QLL-OH
HRMS (ESI): *m/z* calcd. $[C_{57}H_{80}N_9O_{12}SSi]^+$: 1142.5416, found: 1142.5408.

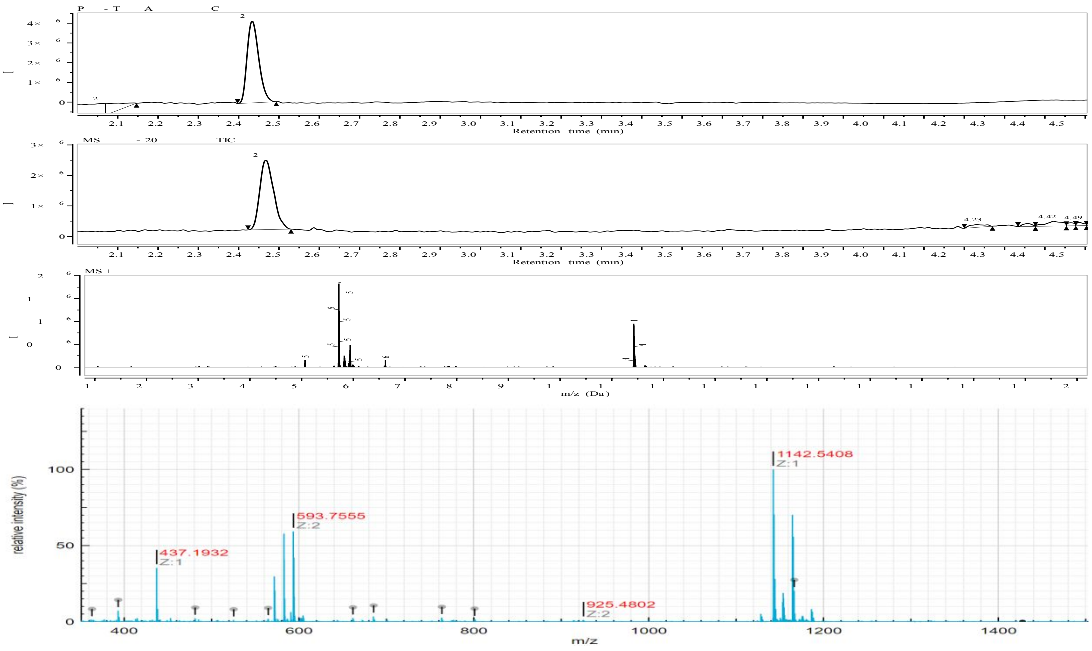

Figure S37. Structure, condensed formula, exact mass and LC-MS traces and extracted peak of peptide **P1**.

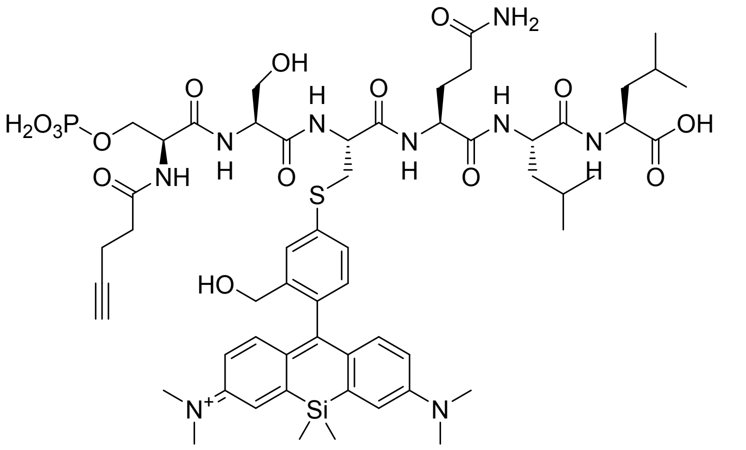

Pentynyl-pSSC(HMSiR)QLL-OH
HRMS (ESI): *m/z* calcd. $[C_{57}H_{81}N_9NaO_{15}PSSi]^{+2}$: 622.7483; Found 622.7492.

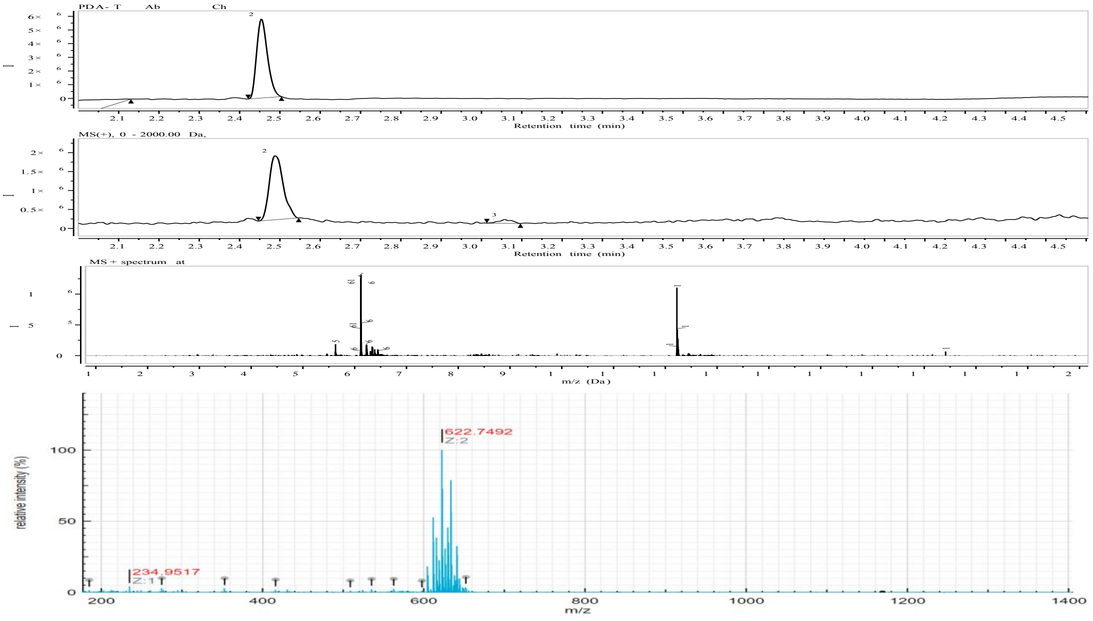

Figure S38. Structure, condensed formula, exact mass and LC-MS traces and extracted peak of peptide **P2**.

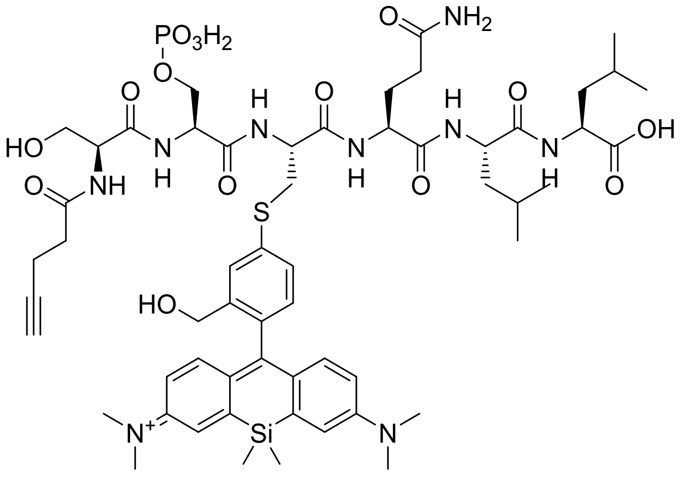

Pentynyl-SpSC(HMSiR)QLL-OH

HRMS (ESI): *m/z* calcd. $[C_{57}H_{82}N_9O_{15}PSSi]^{+2}$: 611.7574, found: 611.7597.

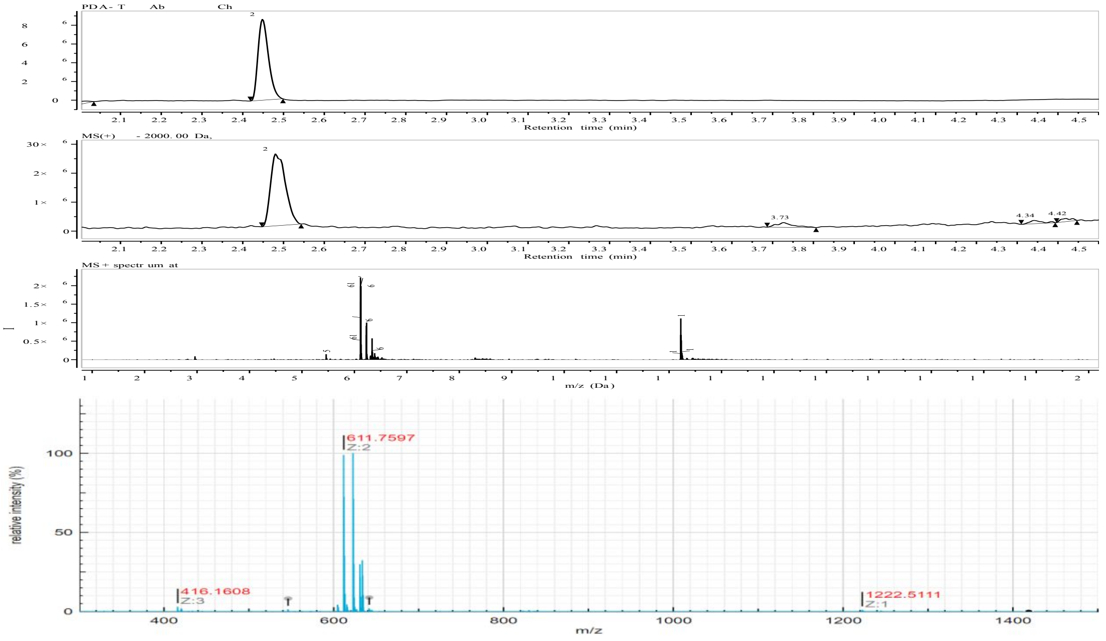

Figure S39. Structure, condensed formula, exact mass and LC-MS traces and extracted peak of peptide **P3**.

4 Supporting Tables

Table S1. Settings applied in the single-molecule localization software Picasso [7]. MLE = maximum likelihood estimation.

Parameter	Value
Box side length	5 px
Fit method	MLE
Gradient	7000
Baseline	100
Sensitivity	15.6
Gain	300
Quantum yield	0.93

Table S2. Filter parameters applied in custom code to remove noisy traces. MLE = maximum likelihood estimation. *Filtering applied only for classical ML.

Parameter	Value
Threshold for peaks (in number of standard deviations)	8
Minimal peak width (in frames)	1
Minimal peak number	10
Difference in #frames between 1st and 2nd peak	MLE
Last frame for a first peak to occur	700*
Fourier transform maximal intensity	<1000

Table S3. A list of features extracted for the correlation plots, PCA and classical ML approaches.

Feature
Number of peaks
Last peak
Blinking time
Maximal peak height
Mean peak height
Std peak height
Total ON time (sum of peak widths)
Maximal ON time (peak width)
Mean ON time (peak width)
Std ON time (peak width)
Total OFF time
Maximal OFF time
Mean OFF time
Std OFF time
Maximal approximate peak area
Minimal approximate peak area
Mean approximate peak area
Std approximate peak area
Tempo[10]

Table S4. Parameters used for the classical ML models. If no specific numbers are mentioned the default settings were used.

Model type	Scikit-learn parameters used
Decision Tree	default
Random Forest	default
AdaBoost	default
KNN	n_neighbors=6
SVM (SVC)	cache_size=1000
MultiLayer Perceptron	alpha=0.05, learning_rate='adaptive', max_iter=1000

Table S5. Summary of the number of traces used for training and testing, as well as the required training times and evaluation time for MC dropout, rounded up to the minute. The models were trained on the ScienceCluster of UZH using a Tesla V100-SXM2-32GB GPU, and the evaluation and analysis of the MC dropout models were conducted on a ScienceCloud virtual machine instance using 8 cores of an AMD EPYV 7702 Hypervisor CPU and an nVidia Tesla T4 GPU (infrastructure services provided by the Science IT team of the University of Zurich, www.s3it.uzh.ch).

Compound	Total # traces	# Traces for training	# Traces for testing	Time for training (without MCD)	Evaluation for MCD analysis
Total for charged set	11896	9517	2379	32 min (12 min)	4 min
C1	3572	2858	714		
C2	1756	1405	351		
C3	4970	3976	994		
C4	1598	1278	320		
Total for phosphorylation set	4162	3330	832	11 min (5 min)	4 min
P1	1176	941	235		
P2	1198	959	239		
P3	1788	1430	358		
Total for epimer set	31504	25204	6300	1 h 23 min (33 min)	4 min
E1	11226	8981	2245		
E2	11388	9111	2277		
E3	8890	7112	1778		

Table S6. Detailed model architecture of the 1D-CNN model with all the parameters that were set manually. Parameters that are not mentioned were used in their default value as set in TensorFlow 2.7.0.

Layer	Layer settings	Param #
Input	input shape=(None, 6000, 2)	
Convolutional layer 1D 1	number of filters=64, kernel size=9, stride size=2, activation="relu"	1216
Batch Normalization	-	256
Dropout	dropout rate=0.3	0
Convolutional layer 1D 2	number of filters=64, kernel size=3, stride size=2, activation="relu"	12352
Batch Normalization	-	256
Dropout	dropout rate=0.5	0
Convolutional layer 1D 3	number of filters=64, kernel size=3, stride size=2, activation="relu"	12352
Flatten	-	256
Dense layer	units=number of classes, activation="softmax"	191492 (for 4 classes)

Table S7. Detailed deterministic model architecture 1D-CNN-GRU with all the parameters that were set manually. Parameters that are not mentioned were used in their default value as set in TensorFlow 2.7.0.

Layer	Layer settings	Param #
Input	input shape=(None, 6000, 2)	
Convolutional layer 1D 1	number of filters=64, kernel size=9, stride size=2, activation="relu"	1216
Batch Normalization	-	256
Dropout	dropout rate=0.3	0
Convolutional layer 1D 2	number of filters=64, kernel size=3, stride size=2, activation="relu"	12352
Batch Normalization	-	256
Dropout	dropout rate=0.5	0
Convolutional layer 1D 3	number of filters=64, kernel size=3, stride size=2, activation="relu"	12352
Batch Normalization	-	256
Dropout	dropout rate=0.3	0
GRU layer	units=128	74496
GRU layer	units=256	296448
Dense layer	units=number of classes, activation="softmax"	771 (for 3 classes)

Table S8. Mean overall accuracies and standard deviations for the classification results on all the 25 models of the five test sets of the five-fold cross-validation (Figure S11) using 1D-CNN-GRU.

Set	Mean overall accuracy	Mean loss
**C1-C4**	0.615450 (±0.020893)	1.306280 (±0.083175)
**P1-P3**	0.687844 (±0.021590)	1.108188 (±0.067480)
**E1-E3**	0.641567 (±0.055850)	1.055840 (±0.025304)

Table S9. The model architecture including MCD in detail with all the parameters that were set manually. Parameters that are not mentioned were used in their default values as set in TensorFlow 2.7.0.

Layer	Layer settings
Input	input shape=(None, 6000, 2)
Convolutional layer 1D 1	number of filters=64, kernel size=9, stride size=2, activation="relu"
Batch Normalization	-
Dropout	dropout rate=0.1
Convolutional layer 1D 2	number of filters=64, kernel size=3, stride size=2, activation="relu"
Batch Normalization	-
Dropout	dropout rate=0.3
Convolutional layer 1D 3	number of filters=64, kernel size=3, stride size=2, activation="relu"
Batch Normalization	-
GRU layer	units=128 with Dropout=0.05
GRU layer	units=256 with Dropout=0.05
Dense layer	units=number of classes, activation="softmax"

5 References

(1) Chollet, F.; others. Keras, 2015.
(2) Martín Abadi; Ashish Agarwal; Paul Barham; Eugene Brevdo; Zhifeng Chen; Craig Citro; Greg S. Corrado; Andy Davis; Jeffrey Dean; Matthieu Devin; Sanjay Ghemawat; Ian Goodfellow; Andrew Harp; Geoffrey Irving; Michael Isard; Jia, Y.; Rafal Jozefowicz; Lukasz Kaiser; Manjunath Kudlur; Josh Levenberg; Dandelion Mané; Rajat Monga; Sherry Moore; Derek Murray; Chris Olah; Mike Schuster; Jonathon Shlens; Benoit Steiner; Ilya Sutskever; Kunal Talwar; Paul Tucker; Vincent Vanhoucke; Vijay Vasudevan; Fernanda Viégas; Oriol Vinyals; Pete Warden; Martin Wattenberg; Martin Wicke; Yuan Yu; Xiaoqiang Zheng. TensorFlow: Large-Scale ML on Heterogeneous Systems, 2015.
(3) Pedregosa, F.; Varoquaux, G.; Gramfort, A.; Michel, V.; Thirion, B.; Grisel, O.; Blondel, M.; Prettenhofer, P.; Weiss, R.; Dubourg, V.; Vanderplas, J.; Passos, A.; Cournapeau, D.; Brucher, M.; Perrot, M.; Duchesnay, É. Scikit-Learn: ML in Python. *J. Mach. Learn. Res.* **2011**, *12*, 2825–2830.
(4) Hunter, J. D. Matplotlib: A 2D Graphics Environment. *Comput. Sci. Eng.* **2007**, *9*, 99–104. https://doi.org/10.1109/MCSE.2007.55.
(5) Waskom, M. L. Seaborn: Statistical Data Visualization. *Journal of Open Source Software* **2021**, *6*, 3021. https://doi.org/10.21105/joss.03021.
(6) Jain, A.; Liu, R.; Xiang, Y. K.; Ha, T. Single-Molecule Pull-down for Studying Protein Interactions. *Nat. Protoc.* **2012**, *7*, 445–452. https://doi.org/10.1038/nprot.2011.452.
(7) Schnitzbauer, J.; Strauss, M. T.; Schlichthaerle, T.; Schueder, F.; Jungmann, R. Super-Resolution Microscopy with DNA-PAINT. *Nat. Protoc.* **2017**, *12*, 1198–1228. https://doi.org/10.1038/nprot.2017.024.
(8) Virtanen, P.; Gommers, R.; Oliphant, T. E.; Haberland, M.; Reddy, T.; Cournapeau, D.; Burovski, E.; Peterson, P.; Weckesser, W.; Bright, J.; van der Walt, S. J.; Brett, M.; Wilson, J.; Millman, K. J.; Mayorov, N.; Nelson, A. R. J.; Jones, E.; Kern, R.; Larson, E.; Carey, C. J.; Polat, İ.; Feng, Y.; Moore, E. W.; VanderPlas, J.; Laxalde, D.; Perktold, J.; Cimrman, R.; Henriksen, I.; Quintero, E. A.; Harris, C. R.; Archibald, A. M.; Ribeiro, A. H.; Pedregosa, F.; van Mulbregt, P.; Vijaykumar, A.; Bardelli, A. P.; Rothberg, A.; Hilboll, A.; Kloeckner, A.; Scopatz, A.; Lee, A.; Rokem, A.; Woods, C. N.; Fulton, C.; Masson, C.; Häggström, C.; Fitzgerald, C.; Nicholson, D. A.; Hagen, D. R.; Pasechnik, D. V.; Olivetti, E.; Martin, E.; Wieser, E.; Silva, F.; Lenders, F.; Wilhelm, F.; Young, G.; Price, G. A.; Ingold, G. L.; Allen, G. E.; Lee, G. R.; Audren, H.; Probst, I.; Dietrich, J. P.; Silterra, J.; Webber, J. T.; Slavič, J.; Nothman, J.; Buchner, J.; Kulick, J.; Schönberger, J. L.; de Miranda Cardoso, J. V.; Reimer, J.; Harrington, J.; Rodríguez, J. L. C.; Nunez-Iglesias, J.; Kuczynski, J.; Tritz, K.; Thoma, M.; Newville, M.; Kümmerer, M.; Bolingbroke, M.; Tartre, M.; Pak, M.; Smith, N. J.; Nowaczyk, N.; Shebanov, N.; Pavlyk, O.; Brodtkorb, P. A.; Lee, P.; McGibbon, R. T.; Feldbauer, R.; Lewis, S.; Tygier, S.; Sievert, S.; Vigna, S.; Peterson, S.; More, S.; Pudlik, T.; Oshima, T.; Pingel, T. J.; Robitaille, T. P.; Spura, T.; Jones, T. R.; Cera, T.; Leslie, T.; Zito, T.; Krauss, T.; Upadhyay, U.; Halchenko, Y. O.; Vázquez-Baeza, Y. SciPy 1.0: Fundamental Algorithms for Scientific Computing in

Python. *Nat. Methods* **2020**, *17*, 261–272. https://doi.org/10.1038/s41592-019-0686-2.
(9) Van Der Walt, S.; Colbert, S. C.; Varoquaux, G. The NumPy Array: A Structure for Efficient Numerical Computation. *Comput. Sci. Eng.* **2011**, *13*, 22–30. https://doi.org/10.1109/MCSE.2011.37.
(10) McFee, B.; Raffel, C.; Liang, D.; Ellis, D.; McVicar, M.; Battenberg, E.; Nieto, O. Librosa: Audio and Music Signal Analysis in Python. In *Proceedings of the 14th Python in Science Conference*; 2015; pp 18–24. https://doi.org/10.25080/majora-7b98e3ed-003.
(11) Kingma, D. P.; Ba, J. Adam: A Method for Stochastic Optimization. *arXiv* **2017**.
(12) Yen, J.-C.; Chang, F.-J.; Chang, S. A New Criterion for Automatic Multilevel Thresholding. *IEEE Transactions on Image Processing* **1995**, *4*, 370–378. https://doi.org/10.1109/83.366472.
